# Series-Spatial Transcriptome Profiling of Leafy Head Reveals the Key Transition Leaves for Head Formation in Chinese Cabbage

**DOI:** 10.3389/fpls.2021.787826

**Published:** 2022-01-06

**Authors:** Xinlei Guo, Jianli Liang, Runmao Lin, Lupeng Zhang, Jian Wu, Xiaowu Wang

**Affiliations:** Institute of Vegetables and Flowers, Chinese Academy of Agricultural Sciences, Beijing, China

**Keywords:** leafy head, transcriptome, key transition leaves, hormone, Chinese cabbage

## Abstract

Chinese cabbage is an important leaf heading vegetable crop. At the heading stage, its leaves across inner to outer show significant morphological differentiation. However, the genetic control of this complex leaf morphological differentiation remains unclear. Here, we reported the transcriptome profiling of Chinese cabbage plant at the heading stage using 24 spatially dissected tissues representing different regions of the inner to outer leaves. Genome-wide transcriptome analysis clearly separated the inner leaf tissues from the outer leaf tissues. In particular, we identified the key transition leaf by the spatial expression analysis of key genes for leaf development and sugar metabolism. We observed that the key transition leaves were the first inwardly curved ones. Surprisingly, most of the heading candidate genes identified by domestication selection analysis obviously showed a corresponding expression transition, supporting that key transition leaves are related to leafy head formation. The key transition leaves were controlled by a complex signal network, including not only internal hormones and protein kinases but also external light and other stimuli. Our findings provide new insights and the rich resource to unravel the genetic control of heading traits.

## Introduction

Chinese cabbage (*Brassica rapa* ssp. *pekinensis*) is a widely cultivated vegetable crop in East Asia. It has a unique product organ, a leafy head, which is a distinct type of plant architecture shared by several vegetable crops, including cabbage, mustard, endive, and lettuce (Wang et al., 2014; Yu et al., 2020). The vegetative growth period of Chinese cabbage can be divided into three stages: seedling, rosette and heading stage (Yu et al., 2013). At the seedling stage, the leaves are round and flat with long petioles, while at the rosette stage, the inner new leaves are continually formed at the shoot apical meristem (SAM) and subsequently become large with short petioles, which start to grow more upright surrounding the axis of the enlarged, but compressed stem (Opena et al., 1998; Sun et al., 2019).

When it comes to the heading stage, some rosette leaves begin to incurve and fold upward. As more new leaves form and expand around the SAM, they become increasingly entrapped until they remain folded in the center to form leafy heads as nutrient storage organs. When leafy heads are ready for harvest, the leaves are wrinkled with broad midveins and broad fleshy petioles (Sun et al., 2019), and different regions of the blade and petiole also show varying degrees of curvature. In addition, various types of leaf morphology can be distinguished from the inner to outer leaves. The inner head leaves (HLs) curve inward, are wrapped by outer HLs, and are not exposed to the sunlight. They can be white, orange, or yellow, while outer HLs are green, grow upright, and curve outward (Li et al., 2012; Li Y. et al., 2019). In contrast, the outermost leaves are non-heading and close to the soil surface and easily become senescent and rotting. Generally, the outer leaves are considered to promote head formation by supplying photosynthates to the inner leaves for head formation, as well as providing shade to the head (He et al., 2000; Wang et al., 2014). Leaf morphological and functional differentiation with the growth stage and spatial position indicate that leafy head formation is a very complex process.

As for the leafy head domestication trait, some genes involved in adaxial–abaxial polarity pathway and hormones related genes, were found to be under selection in leaf-heading accessions, indicating their contribution to leafy head formation (Cheng et al., 2016; Liang et al., 2016; Cai et al., 2021). Genetic analysis has demonstrated that the ad-ab polarity pathway gene *BcpLH* affected the inward curvature of leaves (Yu et al., 2000; Ren et al., 2020). Transcription factors, such as *BrpTCP4* and *BrpSPL9*, have been proven to be involved in leafy head formation by affecting head shape and heading time, respectively (Mao et al., 2014; Wang et al., 2014). In addition, gibberellin biosynthesis related gene *BrKS1* was identified for leafy head formation by screening the EMS mutant library of Chinese cabbage (Gao et al., 2020). Auxin biosynthesis genes (*AUXs*) have also been investigated and demonstrated to be involved in leafy head formation, possibly by regulating the concentration of auxin (He et al., 2000).

In addition to the differences in leaf morphology, the leaf functions are also different; i.e., the rosette leaves act as photosynthetic organs, while leafy head serves as a nutrient sink organ (He et al., 2000). Carbohydrate accumulation in the leaves was also considered to be closely related to leafy head formation (Ito and Kato, 1957; Wang et al., 2012). Sucrose, fructose and glucose are the main soluble sugars, most of which in edible organs come from the photosynthate of source leaves. Sucrose is the main form of photosynthate output, which can be decomposed into fructose and glucose in sink cells (Park et al., 2008). The distribution and composition of carbohydrates in sink tissues depends on the relative gene expression of enzymes in carbohydrate metabolism. A recent study investigated differing sugar accumulation and expression patterns of sugar-metabolizing enzyme genes in heading leaves of Chinese cabbage, and then identified the important role of sucrose synthase (SUS1) in leafy head formation (Liu et al., 2021).

To reveal the molecular mechanism underlying leafy head formation, a range of transcriptomic studies were performed based on the comparison of changes in transcript abundance of only certain leaves at different development stages, or between heading Chinese cabbage or non-heading pak-choi (Wang et al., 2012; Li J. et al., 2019; Sun et al., 2019). Wang et al. (2012) discovered that some stimuli (such as carbohydrate levels, light, and hormones), and transcription factors, protein kinases and calcium, may play important roles in leafy head development. In addition, hormones such as auxin, cytokinin, abscisic acid, gibberellin, and brassinosteriods (BR), were found to perhaps play important roles in leafy head formation (Gao et al., 2017; Gu et al., 2017; Li J. et al., 2019). However, previous transcriptome analyses to date have focused on only the inner leaves between different stages or simply separated the blade and midrib of internal and external leaves of leafy heads, which ignores the various leaf morphologies at heading stages that may play diverse and potentially fundamental roles in leafy head formation. To date, the genetic basis and molecular mechanism of leafy head formation remain largely unclear, including the roles of leaf blades and petioles.

Considering the significant differences in leaf morphology, including age, color, size, and curvature degree, the inner to outer leaves of leafy head at the heading stage reflect their functional diversification and specialization; i.e., the outer leaves might act as source tissues, while the inner leaves act as sink tissues. In addition, even on the same leaf, the different regions show significantly different structures and curvature degrees, indicating their different contributions to the leaf heading process. Here, we investigated the transcriptome profiles of continuous head leaves from inside to outside at the heading stage, including inner SAM, inner leaves (covered and not exposed to sunlight), and outer leaves (exposed to sunlight), which represented different leaf morphology. Moreover, five different regions in the blade and petiole of head leaves were further separated considering that different regions on the same head leaf show different degrees of curvature. The series-spatial profiles of genome-wide gene expression were presented in a total of 24 different leaf tissues, identifying the key transition head leaves that likely have an important role in leaf-heading. Overall, our study provided new insights into leafy head formation.

## Results

### RNA-Seq of Dissected Leaves at the Heading Stage of Chinese Cabbage

**FIGURE 1:**
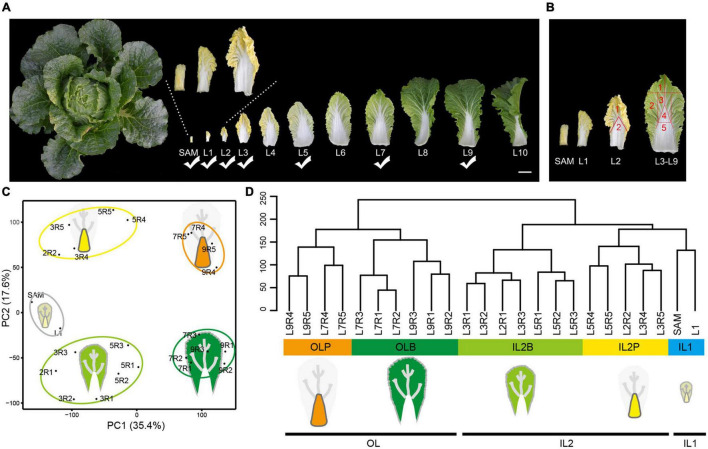
Transcriptome profiling of Chinese cabbage leaves in different leaf layers at the heading stage. **(A)** Representative pictures of leaves spanning 11 leaf layers. The leaf layers marked with a check mark were used for RNA-seq. Scale bar is 5 cm. **(B)** L2 was divided into two regions (including leaf blades and petioles) for sampling. L3, L5, L7, and L9 were divided into five regions (named R1, R2, R3, R4, and R5, respectively) for sampling. **(C)** PCA of the transcriptomes of the 24 different tissues. The first PC (PC1) distinguished leaf positioning from SAM and explained 35.4% of the total sample variation. The second PC (PC2; 17.6% of sample variance) clearly distinguished the petioles from the leaf blades. **(D)** The cluster dendrogram for total samples. IL1 inner leaves 1; IL2B, blade of inner leaves 2; IL2P, petiole of inner leaves 2; OLB, outer leaf blade; OLP, outer leaf petiole.

The leafy head formation of Chinese cabbage is attributed to significantly spatial leaf morphological differentiation. The head leaves presented two regions, the blade and petiole, which show different structures and curvature degrees. The head leaves from the inside to the outside also showed significant morphological variations (Figure 1A). In addition to the gradually increasing leaf size, the color of the leaf blades changed from yellow (L1–L4) to yellow–green (L5–L6), then became light green (L7) and dark green (L8–L9). Moreover, the SAM and inner leaves (L1–L6) curved inward, while the outer leaves (L8–L9) curved slightly outward. Of all the leaves, L7 was positioned at the boundary between the inner incurved leaves and outer outward-curved leaves, and they covered all the inner leaves and provided shade to form the head. Here, L7 was defined as an outer leaf.

To explore the molecular processes underlying the leafy head formation, comprehensive transcriptome analysis was conducted on 24 leaf tissue samples with two biological replicates for each tissue, including the shoot apical meristem with the incipient youngest leaves (SAM), whole inner leaves (L1), and dissected blade and petiole tissues from three inner leaves (L2, L3, and L5), and two outer leaves (L7, L9), which represented significant differences in leaf morphology (Figures 1A,B). Transcriptome sequencing yielded 7.43 × 10^8^ high-quality clean reads from 48 samples (1.549 × 107 reads per sample, on average; Supplementary Table 1). All biological replicates showed a high correlation, with a Pearson correlation coefficient of 0.92–0.99 (Supplementary Figure 1). After mapping clean reads to the Chinese cabbage reference genome, transcripts per million (TPM) was calculated for each gene. A total of 27,876 expressed genes were identified (Supplementary Figure 2A and Supplementary Table 2), and 75.08% of genes (20929/27876) were commonly expressed in all samples (Supplementary Figure 2B).

Principal component analysis (PCA) showed a clear separation along principal components (PC) 1 and 2. PC1 separated the leaf samples according to their distance from the SAM, whereas PC2 separated the blade and petiole tissues. In combination with hierarchical clustering analysis, the transcriptomes were divided into five distinct leaf tissue groups. Samples from SAM and L1 formed inner leaves 1 (IL1) and represented the tissues that were closest to the shoot apex. Blades of inner leaves 2 (IL2B) and petioles of inner leaves 2 (IL2P) represented the blade and petiole tissues of L2–L5, whereas the outer leaf blade (OLB) and outer leaf petiole (OLP) represented the blade and petiole tissues of outer leaves (L7 and L9), respectively (Figures 1C,D). These groups were in accordance with our observation of leaf morphological differentiation in these selected leaf samples, indicating that our sampling strategy would identify tissue-specific genes from different head leaves.

### Profiling of Differentially Expressed Genes in Heading Leaves

**FIGURE 2:**
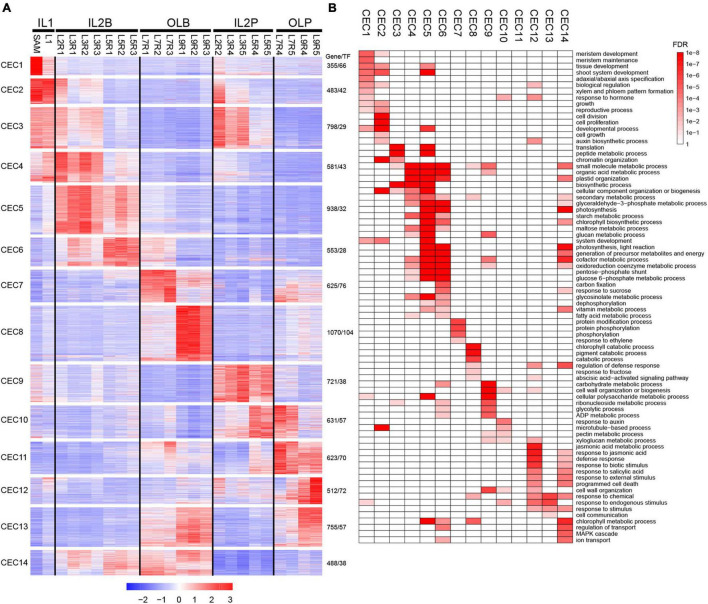
Expression profiles and functional transition of 9,133 DEGs in different leaves. **(A)** K-means clustering of DEGs. A total of 9,133 DEGs were grouped into 14 clusters. For each gene, expression data were Z-score standardized. **(B)** Gene ontology (GO) slim categories of each co-expressed cluster were presented as a heatmap with a color gradient based on the false discovery rate (FDR) values. Only significant GO categories (FDR < 0.05) were displayed.

Differentially expressed genes (DEGs) were identified in different leaves at the whole-genome level to explore the diverse and potential functions among these leaves. A total of 9,133 DEGs were found, including 5,186 DEGs among leaf blades and 6,136 DEGs among petioles, as well as 5,514 DEGs between leaf blades and petioles (Supplementary Figure 3). A display of DEGs (Figure 2A) showed that they were divided into 14 co-expression clusters (CEC1–CEC14; Supplementary Figure 4) using the k-means clustering, and these CECs were closely related to the five groups of HL samples in Figures 1C,D.

The high expression level of DEGs in IL1 was best represented by CEC 1 and 2 (Figure 2A and Supplementary Table 3). CEC 1 was highly expressed in SAM and was represented by genes related to meristem development, tissue development, and adaxial/abaxial polarity (Figure 2B). For example, *STM*, *KNAT2*, and *KNAT6* in SAM are required to maintain indeterminate cell fate and to prevent cell differentiation in the meristem (Carles and Fletcher, 2003). In addition, *BOP1* and *BOP2* are expressed in the proximal region to directly activate the adaxially expressed *AS2* and the boundary-expressed *LOB* (Jun et al., 2010). CEC 2 was highly expressed at SAM and L1 and contained a set of genes related to cell division, cell proliferation, cell growth, and auxin biosynthetic process (Figure 2B), such as *CDKB1*, *CDKB2*, *CYCA1;1*, *AN3*, *ANT*, *FKD1*, and *PIN1*. Previous studies showed that *CDKBs*, *CYCAs*, *ANT*, and *AN3* were involved in cell proliferation during leaf development (Powell and Lenhard, 2012; Kalve et al., 2014). In developing leaf veins, FORKED1 is required for localization of PIN1, contributing to organ positioning, separation, and outgrowth (Vernoux et al., 2000; Hou et al., 2010).

The high expression level of DEGs in IL2B was best represented by CEC 4–6 (Figure 2A and Supplementary Table 3). CEC 4, 5, and 6 were all enriched in the organic acid metabolic process, starch metabolic process, plastid organization, and glycosinolate metabolic process. Additionally, both CEC 5 and 6 contained substantial expression of genes related to photosynthetic light reactions, as well as several key genes of the Calvin cycle, including *PSAE2*, *PSBQ2*, *CAB*, *PSAK*, and *PSAL* (Figure 2B). The high expression level of DEGs in IL2P was best represented by CEC 9 (Figure 2A and Supplementary Table 3). The CEC 9 were enriched in plant cell wall-related process, including wall organization or biogenesis, and pectin metabolic processes (Figure 2B). For example, *IRX9*, *IRX3*, and *IRX15* are essential for normal xylan synthesis and deposition in the secondary cell wall (Doering et al., 2012). In addition, the genes encoding expansins (*EXPA3*, *EXPA4*, *EXPA9*, and *EXP12*) were also enriched in CEC 9 (Kalve et al., 2014).

The high expression level of DEGs in OLB was best represented by CEC 7 and 8 (Figure 2A and Supplementary Table 3). CEC 7 were highly expressed in leaf blade of L7 and were represented by genes related to protein modification process, protein phosphorylation, and protein serine/threonine kinase activity (Figure 2A), such as *BRI1*, *CPK5*, and *CRK39*. These results reflected that the L7 was characterized by obvious signal transduction and gene regulation characteristics. CEC 8, with a high expression level in the leaf blade of L9, contained a set of genes related to chlorophyll catabolic process, cellular catabolic process, regulation of defense response, response to fructose, and abscisic acid-activated signaling pathway (Figure 2B). For example, *MCCA*, *ACD1*, *ACD2*, and *HGO* were involved in the catabolic process (Ding et al., 2012; Pattanayak et al., 2012; Han et al., 2013). Abscisic acid (ABA) regulates various developmental processes and adaptive stress responses in plants (Cutler et al., 2010). In addition, many abscisic acid-responsive genes were also enriched in CEC 8, including *ABI5*, *ABI1*, *SNRK2*, *CIPK15*, *NAC019*, *ANAC2*, *PYL7*, and *MYB74*.

The high expression level of DEGs in OLP was best represented by CECs 11 and 12 (Figure 2A and Supplementary Table 3). The genes in CEC 11 were highly expressed in the petioles of L7–L9, but they were not enriched in any Gene ontology (GO) term. The genes in CEC 12 were highly expressed in the petiole of L9. These genes might be mainly involved in the abscisic acid-activated signaling pathway, jasmonic acid (JA) metabolic process, JA responsion, defense responsion, biotic stimulus responsion, salicylic acid responsion, and external stimulus responsion. Many JA pathway genes were enriched in CEC 12, including JA biosynthetic genes (AOC3, OPR3, JASSY, AOS, and LOX4) and JA response genes (MYBR1, ILL6, TAT3, JAZ1, JAZ5, JAZ6, MYC2, JAZ10, JAZ8, and JAZ3) (Huang et al., 2017; Wasternack and Song, 2017). These results suggest that L9 may play a role in protecting the leafy head from external damage.

Although whole-genome transcriptomic profiling suggested five distinct groups for leaf samples (Figures 1C,D), a total of 3,051 highly expressed genes, including 195 TFs, in CECs 3 and 4, CECs 6 and 14, and CEC 10 may have similar functional processes in IL1-IL2B, IL2B-OLB, and IL2P-OLP, respectively, as these genes represented similar expression patterns between groups (Figure 2A and Supplementary Table 3). These shared genes may have important roles in the functional transition from one group to another. The discovery of group-specific and group-shared highly expressed genes indicated the dynamic expression profiling existing in heading leaves.

### Expression of Genes Related to Leaf Development Reveals the Key Transition Leaves in Leafy Head

**FIGURE 3:**
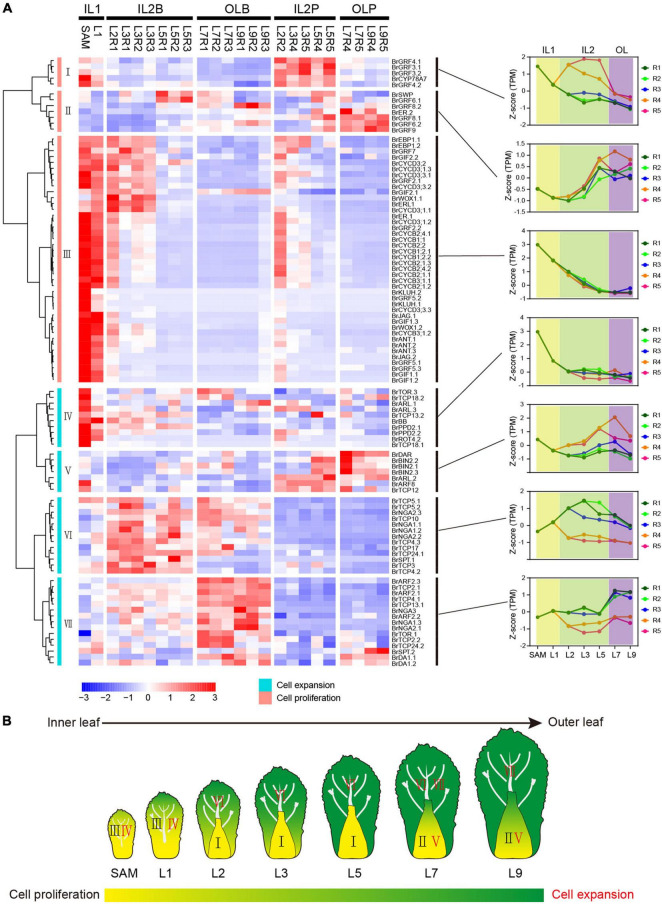
Expression pattern of cell proliferation and cell expansion genes. **(A)** Hierarchical clustering analysis of cell proliferation genes and cell expansion genes. For each gene, expression data were Z-score standardized. **(B)** Expression model of cell proliferation genes and cell expansion genes in Chinese cabbage at the heading stage.

Cell proliferation and elongation rates along different axes directions shape leaf morphology (Tsukaya, 2003; Horiguchi et al., 2006; Nakata and Okada, 2013). We identified previous characterized cell proliferation-related genes and cell expansion-related genes (Powell and Lenhard, 2012; Du et al., 2018; Supplementary Table 4 and Supplementary Figure 5), to investigate the role of cell proliferation and expansion genes in morphology differentiation of head leaves. We found that most cell proliferation genes peaked in IL1 HLs, including SAM and L1, but were rapidly downregulated in IL2 HLs and outer HLs (Figure 3A), implying their important role in the initiation of the constantly growing inner leaves. Unlike cell proliferation genes, most cell expansion genes were highly expressed in the blade regions of IL2 HLs and outer HLs, and they displayed more diverse expression patterns and were divided into four clusters (Figure 3A). Notably, several genes in cluster VI had higher expression levels in R1 and R2 than in R3, such as *BrTCP5.1*, *BrTCP5.2*, *BrNGA1.1*, and *BrNGA1.2*, indicating their potential role in leaf marginal growth of HLs. The genes from cluster V were dominantly expressed in the petioles, indicating that they have a specific role in petiole growth. Furthermore, genes from clusters V, VI, and VII showed significantly different patterns between IL2 HLs and outer HLs. Cluster VI genes showed significant downregulation in outer HLs compared with IL2 HLs, while cluster VII genes dominantly expressed in outer HLs than IL2 HLs and cluster V genes showed the highest expression in the outer HL L7. These results indicated that L7 are special transition leaves, which was in accordance with our observation that L7 were positioned as key boundary leaves between the outer and inner leaves. Overall, our results suggested that leafy head growth included a developmental transition of cell proliferation-dominant in IL1 HLs to cell expansion-dominant in IL2 and outer leaves, and cell division/cell expansion of the leaf blade and petiole were coordinated by different genes. In addition, most cell expansion genes showed different expression pattern between IL2 HLs and outer HLs, resulting in a significant change in the regulation of cell division and cell expansion in L7 compared with IL2 HLs and outer leaves (Figure 3B).

**FIGURE 4:**
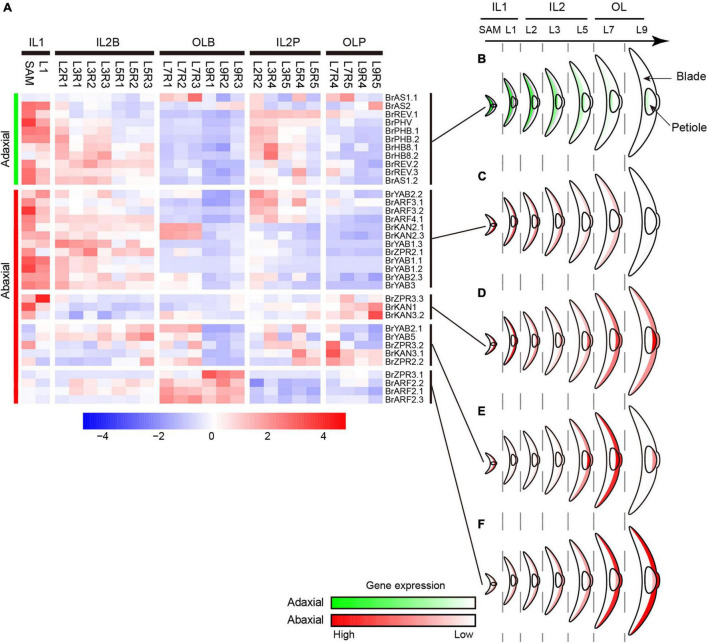
Expression profiles of ad-ab polarity genes. **(A)** Expression characteristics of ad-ab polarity genes in leafy head. For each gene, expression data were Z-score standardized. **(B)** Expression pattern of adaxial genes. **(C–F)** Four expression patterns of abaxial genes. Crescent indicated transverse section of a leaf.

The curvature of Chinese cabbage HLs is generally considered to be caused by the asymmetric cell growth between the adaxial and abaxial axis of the leaf (Cheng et al., 2016; Liang et al., 2016; Yu et al., 2020). To explore the role of ad-ab polarity genes in the curvature of head leaves, their expression patterns were analyzed (Supplementary Table 5). Almost all adaxial genes, except *BrAS1.1*, showing a significantly low expression level in the outer HLs (Figures 4A,B). For the abaxial gene, two major expression patterns were observed. Many abaxial genes, except *BrKAN2.1* and *BrKAN2.3*, showed a similar expression pattern with adaxial genes; they were predominantly expressed in inner HLs, downregulated from the inner to the outer leaves, and significantly repressed in the outer HLs (Figures 4A,C). Conversely, there were still many abaxial genes were upregulated from the inner to the outer leaves, and significantly upregulated in the outer HL L7, such as *BrARF2.1*, *BrARF2.2*, and *BrARF2.3* (Figures 4D–F). Interestingly, the adaxial gene *BrAS1.1* and two abaxial genes *BrKAN2.1* and *BrKAN2.3* were specifically upregulated in L7. A homologous gene of *AS1* (*LsAS1*) in lettuce has been confirmed to be involved in leafy head formation (Yu et al., 2020), whereas *BrKAN2.1* and *BrKAN2.3* were demonstrated to be under strong selection in heading Chinese cabbage (Cheng et al., 2016). Altogether, most ad-ab polarity genes showed significantly different expression patterns in the outer HLs compared with inner HLs.

Considering the morphological observations, PCA and clustering analysis, and expression of genes related to cell division/expansion and ad-ab polarity, L7 were positioned at the boundary between the inner leaves and outer leaves. L7 curved inward, thus showing a similar characteristic to inner leaves. However, L7 covered all inner leaves, providing shade for inner leaves, but the top of the blades exposed to light were light-green, thus showed similar to outer leaves. Therefore, L7 were classified into the outer leaves group by PCA and clustering. In agreement with this observation, expression analysis of genes related to cell division/expansion and ad-ab polarity revealed that L7 were a transition state showing three patterns. First, L7 showed a similar expression pattern to adjacent inner leaves L5 but were different from adjacent outer L9. Second, L7 showed a similar pattern to the outer L9 but were different from the inner L5. Third, L7 showed a specific pattern compared to the adjacent inner L5 and outer L9. In summary, our results confirmed that L7 and adjacent leaves L5 showed consecutive transition states and acted as transition leaves, but L7 were the key transition leaves.

### Analysis of Sugar Metabolism Further Demonstrates the Key Transition Leaves in Leafy Head

In addition to the differences in leaf morphology between the inner and outer leaves at the heading stage, at this stage, the inner leaves are shielded from light, acting as storage organs, whereas the outer leaves are green and exposed to light, implicating their ability to photosynthesize to provide a carbon source for head development (He et al., 2000). Sucrose is the main form of photoassimilate transportation from the photosynthetic “sources” to the heterotrophic “sinks” (Fu et al., 2011; Chen et al., 2012). Here, we further identified and analyzed the accumulation of soluble sugar in HLs and expression characteristics of related gene (Supplementary Table 6).

**FIGURE 5:**
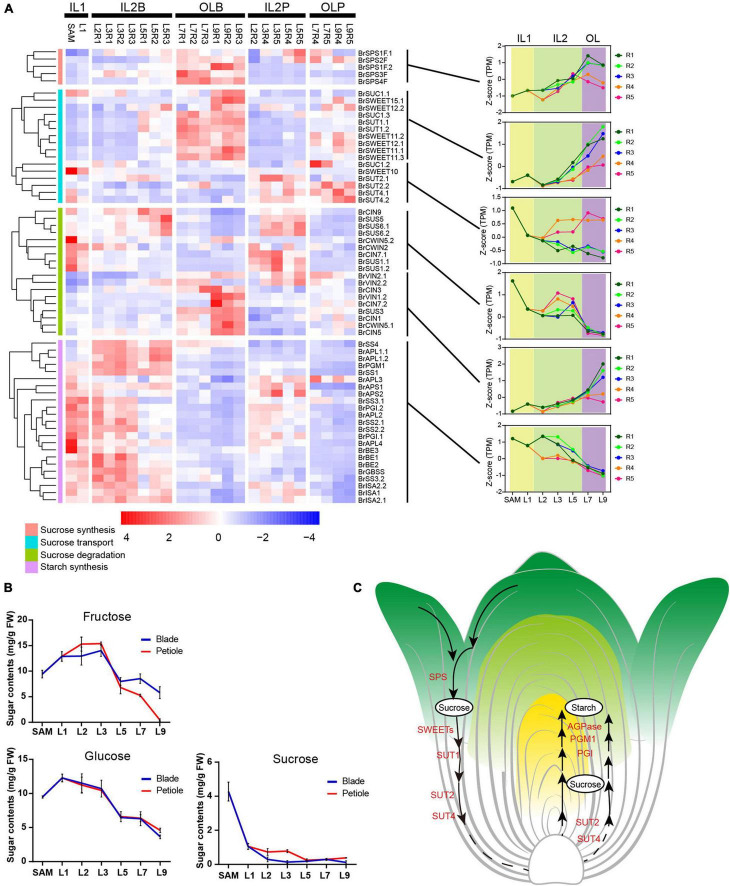
The expression pattern of starch, sucrose and transport genes in leafy head. **(A)** Expression profiles of the genes related to sucrose synthesis, transport, degradation, and starch synthesis. For each gene, expression data were Z-score standardized. **(B)** Soluble sugar content in different leaves. The X axis depicts 12 samples, and the y axis depicts the sugar content. Error bars represent the SD (n = 2). **(C)** A model proposed for sucrose synthesis, transport, degradation and starch synthesis in leafy head.

Almost all genes encoding sucrose-phosphate synthase (SPS), a key enzyme of sucrose synthesis and its activity dictating the identities of source tissues (Ruan, 2014; Rodrigues et al., 2020), were predominantly expressed in outer leaves L7 and L9 (Figure 5A). Consistent with this, several key sucrose transporters (*BrSWEET11s*, *BrSWEET12s*, and two *BrSUT1*) were also preferentially expressed in outer leaves L7 and L9 (Figure 5A). In *Arabidopsis*, *AtSWEET11* and *AtSWEET12* are highly expressed in source leaves and are co-expressed with genes involved in sucrose biosynthesis and phloem loading (Gottwald et al., 2000; Chen et al., 2012). These results indicated that the outer leaves from L7 to L9 were the sites of sucrose synthesis. Interestingly, four genes encoding sucrose transporters (*BrSUT2.1*, *BrSUT2.2*, *BrSUT4.1*, and *BrSUT4.2*) were highly expressed in the petioles of HLs (Figure 5A). According to previous reports, *SUT2* and *SUT4* showed higher expression in sink cells and tissues (Meyer et al., 2004; Schulz et al., 2011; Schneider et al., 2012). These seemed to indicate that the petioles may be sink tissues. Almost all genes encoding SUS, which is an important sucrose-degrading enzyme hydrolyzing sucrose to fructose and UDP-glucose and considered a marker for sink strength (Ruan, 2014), were preferentially expressed in the inner HLs (SAM to L5) (Figure 5A), while other sucrose degrading genes such as *BrCIN3*, *BrCIN7.2*, and *BrCIN1*, showed significantly higher expression in the outer HLs than inner HLs. In addition, starch is an important storage substance in sink organs, and its important synthetic genes were highly expressed in inner HLs (SAM to L5), while lowly expressed in outer HLs. These results also supported that heading leaves from SAM to L5, as well as all petioles, could be storage organs for storing nutrients.

We further determined the content of sucrose, fructose and glucose in different head leaves. In accordance with the expression pattern of *SUSs*, the trend curves of fructose and glucose content in different head leaves were shown as inverse V shapes (Figure 5B). The content of fructose increased dramatically from L1 to L2, peaked at L3, and tapered off from L5–L9, and glucose content was maintained at a high level in the inner HLs (SAM to L3), decreased rapidly from L5 to L9. While the content of sucrose was constantly decreased from inner to outer HLs (Figure 5B). Taken together, our results demonstrated that outer leaves were source tissues that provide energy, while inner leaves the sink tissues for storing nutrients, and the L7 were the key transition leaves for leafy head development (Figure 5C).

### The Key Transition Leaves Are Related to Leaf Heading

**FIGURE 6:**
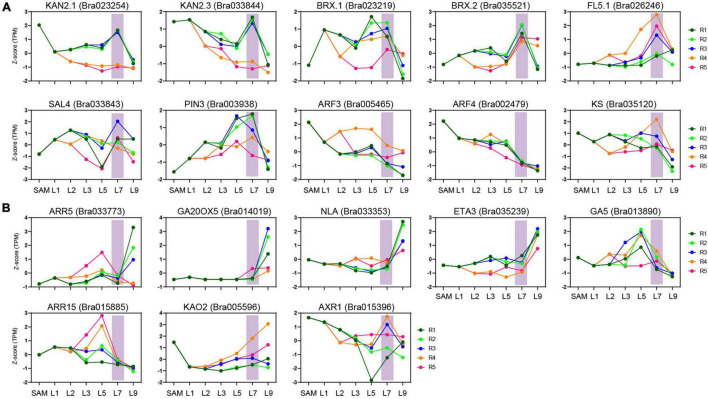
Expression characteristics of heading candidate genes. **(A)** The expression characteristics of reported heading candidate genes in key transition leaves. **(B)** The expression characteristics of heading candidate genes newly identified by domesticated selection analysis.

To explore the contribution of the key transition leaves in leafy head formation, we next analyzed the expression characteristics of the previously identified candidate genes for leafy head formation. Since heading is the result of artificial domestication (Cheng et al., 2016; Zhang et al., 2019), the genes under strong selection in heading *B. rapa* might be candidate genes involved in leafy head formation. In this study, besides ten reported heading-related genes, 13 other leaf-heading candidate genes were further discovered in the domesticated selection region collected from our previously published data (Supplementary Table 7; Cheng et al., 2016; Cai et al., 2021). Surprisingly, nine of the ten reported heading-candidate genes were obviously activated or inhibited in the key transition leaves L7, including two downregulated genes (*ARF3.1* and *ARF4.1*) and seven upregulated genes (*KAN2.1*, *KAN2.3*, *BRX.1*, *BRX.2*, *BrPIN3.3*, *BrFL5.1*, and *BrSAL4.2*) (Figure 6A and Supplementary Table 7). Moreover, eight of the 13 newly identified candidate genes also showed specific expression trends (Figure 6B and Supplementary Table 7). The expression level of *BrKS* (a reported heading-related gene) in the key transition leaves and inner leaves was significantly higher than that of the outer L9, and peaked at the petiole R4 region of the key transition leaves (Figure 6A). The significantly different expression of the heading candidate genes in the key transition leaves compared with other leaves confirmed that these genes will no doubt be the targets of future functional studies, and suggested that the transition leaves were related to leafy head formation.

### Weighted Gene Coexpression Network Analysis Reveals the Complex Signal Interactions That Integrate Phytohormones-External Stimuli in the Transition Leaves

**FIGURE 7:**
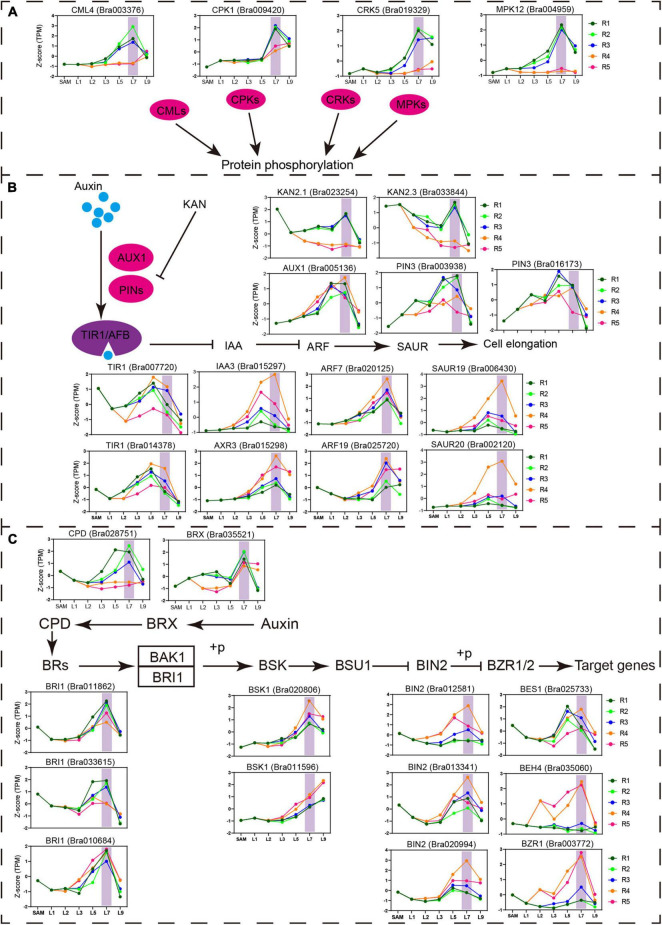
Important pathway identified in the key transition leaves. **(A)** Expression profiles of the genes related to protein phosphorylation and calcium ion binding identified in key transition leaves. Vertical shade boxes indicate transition leaves position. **(B)** Expression profiles of auxin signaling genes upregulated in key transition leaves. Vertical shade boxes indicate transition leaves position. **(C)** Expression profiles of BR signaling genes upregulated in key transition leaves. Vertical shade boxes indicate transition leaves position.

To identify the pathways contributed to the special state of the transition leaf, weighted gene coexpression network analysis (WGCNA) were performed. A total of 17 modules were identified by WGCNA (Supplementary Figure 6). Among them, five modules (greenyellow, magenta, purple, turquoise, and yellow) displayed close associations with transition leaves. Two modules, greenyellow and magenta, were specifically related to the transition leaves and contained many genes encoding protein kinase, including mitogen-activated protein kinases (MAPK), calcium-dependent protein kinases (CDPKs), and cysteine-rich receptor-like kinases (CRKs) (Figure 7A, Supplementary Figures 7, 8, and Supplementary Table 8). These protein kinases are key actors in plant signaling and play an important role in plant development, and external stimulus and phytohormone responses (Xu and Zhang, 2015; Baba et al., 2018; Shi et al., 2018). For example, MPK12 is a negative regulator of auxin signaling, and its kinase activity is increased following auxin treatment (Lee et al., 2009). In addition, the activity of MAPKs is also regulated by BR signals (Kim et al., 2012). In *Arabidopsis*, AtCRK5 can phosphorylate the hydrophilic loops of PIN3, impacting auxin distribution (Ding et al., 2011; Baba et al., 2019). AtCRK1 regulates the light response, and the Atcrk1-1 mutant shows serious growth defects under continuous illumination (Baba et al., 2018). Here, from the inner to outer leaves, *CRK1*, *CPK5*, *MPK12*, and *CML4* displayed increased expression in the blades of L7 but rapidly decreased in L9, indicating that key transition leaves L7 might be regulated by complex signal interactions, not only light and other external stimuli, but also internal hormones.

As expected, multiple phytohormonal pathways were activated in the petioles or blades of the transition leaves (Figures 7B,C and Supplementary Figure 7). Many auxin signaling pathway genes *PIN3*, *TIR1*, *AXR3*, *ARF7*, *ARF19*, *SAUR20*, and *SAUR19* were highly expressed in key transition leaves L7 (Figure 7B). As reported, *ARF7*, *ARF19*, and *SAUR19* constituted a module that mediates the bending growth of hypocotyls in response to light and gravity stimulation (Wang et al., 2020). The icu6, semi-dominant mutation of *AXR3*, results in leaf incurvature (Pérez-Pérez et al., 2010). Meanwhile, *AXR3* could be induced by exogenous BR and is significantly downregulated in bri1 plants (Kim et al., 2006; Nakamura et al., 2006). Many BR signaling pathway genes showed predominant expression in transition leaves, including synthesis gene *CPD*, receptor gene *BRI*, and signal transduction genes *BSK1*, *BIN2*, *BES1*, *BZR1*, and *BEH4* (Figure 7C). The mutation of *BRI1* resulted in shorter petioles and curled leaves in *Arabidopsis* (Yang et al., 2011). BRX mediates the feedback regulation loop between BR synthesis and auxin signaling (Mouchel et al., 2006; Scacchi et al., 2009; Marhava et al., 2018). Overexpression of Chinese cabbage *BrBRX.2* in *Arabidopsis* caused curling leaves with changes in leaf ad–ab polarity patterning (Zhang et al., 2021). Those results indicated that the crosstalk between auxin and BR may play an important role in key transition leaves.

In addition, a lot of light-responsive genes were also activated in transition leaves, such as *FHY3*, *DAG2*, *RAX2*, *COP1*, *PHOT1*, and *PHYB* (Supplementary Figure 8). COP1 is a central repressor of photomorphogenesis and directly interacts with BIN2 and modulates its kinase activity (Ling et al., 2017). PHOT1 is a blue-light photoreceptor that regulates leaf development and morphology by mediating auxin efflux (Jenness et al., 2020). Some important transcription factors that regulate leaf development were also identified, including PIF4, TCP24, TCP2, MYB30, HB12, BLH2, BrKAN2.1, and BrAS1.1, were identified (Supplementary Figure 8). MYB30 is closely integrated with the phytochrome-PIF4/PIF5 signaling module to participate in photomorphogenesis (Yan et al., 2020). Collectively, our data show that a complex signal network integrates plant hormones and external stimuli to maintain the special state of transition leaves.

## Discussion

To explore the mechanism of leafy head formation, many transcriptome studies of Chinese cabbage leaves have been conducted (Wang et al., 2012; Li J. et al., 2019; Sun et al., 2019). However, most heading candidate genes discovered through domestication selection analysis, genetic analysis, or molecular biology have not been identified by these studies. The reason may be that previous transcriptome studies only focused on a certain leaf between different stages, or analyzed mixed-leaf samples, thereby ignoring the diverse and potentially fundamental roles of complex head leaves with different morphology during leaf heading and limiting the value of transcriptome data to understand the process of leafy head formation. In this study, we constructed a spatial transcriptome landscape of leafy head using 24 dissected leaf tissues at the heading stage. Our samples contained continuous head leaves representing very different leaf morphologies. For example, the outer leaves wee yellow-green or green, and curved outward, while the inner leaves were covered by outer leaves, showing yellow and inwardly-curved. And the different part of the leaves by dissecting different regions from blades and petioles due to their structure or curvature degree is different. Our spatial sampling approach enabled the detection of tissue-specific or low abundance genes and their spatial expression changes in a leafy head. For example, the previously identified leaf head related genes such as *BrKAN2.1* showed enrichment in blades, while *BrARF3.1* was predominantly expressed in petioles. More importantly, it enabled the identification of the key transition leaves, because most of the genes involved in leaf development, including the genes related to cell division/expansion and ad-ab polarity, and the genes involved in sucrose metabolism showed significant expression changes in the key transition leaves (L7) or in both the key transition and their adjacent leaves (L5).

**FIGURE 8:**
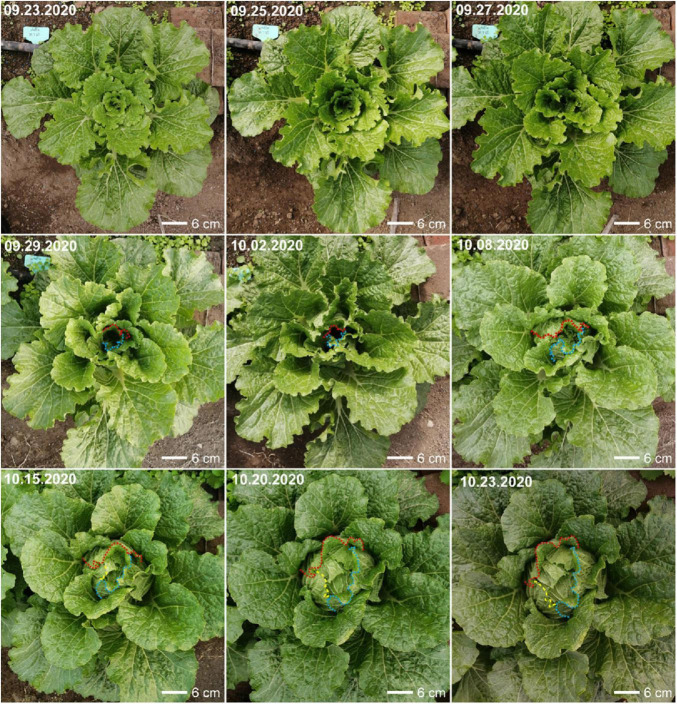
Morphological changes of Chinese cabbage (*Brassica rapa* cv Chiifu-401-42) from rosette stage to heading stage. The edges of key transition leaves were marked by colored dotted lines.

Surprisingly, most heading candidate genes (17/23), including those previously identified and those newly identified by domesticated selection analysis, indeed showed very different expression in the key transition leaves. In addition, an independent experiment was conducted to observe the morphological changes of transition leaves from the rosette stage to the heading stage (Figure 8). At the rosette stage, the new leaves were continually formed at the SAM, and the rosette leaves started to grow more upright. The leaves were arranged as a spiral path. As plants gradually grew up, the fifth whorls of leaves from outside to inside began to curve inward gradually until folding upward, thus covering all the inner leaves and finally forming a leafy head (Figure 8), which were similar to the key transition leaves (L7, the fourth whorls of leaves from outside to inside) identified from the transcriptome analysis. Conversely, the first fourth whorls of leaves, including the outermost leaves that were totally covered, did not curve inward and kept growing upright, resulting in the gentle outward curved in the top of their blades. Our observations showed the key transition leaves as the first inwardly-curved leaves that subsequently folded upward, which covered all the inner leaves and supported a frame for leafy head formation. These results strongly support the existence of special transition leaves, that play very important roles in leafy head formation, suggesting that the key transition leaves are worthy of attention in future heading research.

Accordingly, we further analyzed the specific transcriptional characteristics of the transition leaves through WGCNA. The transition leaves showed the enrichment of transcripts associated with protein kinases, auxin and BR pathways, and the light-responsive pathway, which was consistent with a previous study (Wang et al., 2012). Moreover, many of the previously identified heading-related genes were involved in the auxin and BR signaling pathways, including *ARF3*, *ARF4*, *PIN3*, and *BRX*. Interestingly, the protein kinases that play an important role in cell signal transduction (Xu and Zhang, 2015; Shin et al., 2016) were preferentially highly expressed in the leaf blade of the key transition leaves. Comparatively, many auxin and BR signaling genes were enriched at the R4 position of the key transition leaves, which were located at the top of the petiole and connected to the leaf blade and the petiole (Figure 7). Gao et al. (2017) found an uneven distribution of auxin, and the level of auxin peaked at the lower area of the leaf blades in the HLs in the early folding stage, which was caused by the polar transport of auxin. These results suggested that the transition leaves first curved inward, resulting from complex signal network regulation, including internal hormones, such as auxin and BR, as well as external stimuli, such as light. In addition, the leaf blade and leaf petiole showed different contributions to these signal integrations, and the region R4 is a site worthy of future attention for hormone content analysis.

In summary, the spatial transcriptome of the Chinese cabbage leafy head enabled the identification of the key transition leaves and provided a new perspective for leaf heading in Chinese cabbage. The values of the key transition leaves were illustrated to elucidate the genetic control of the complex leaf morphological and functional differentiation underlying leaf head formation. It also provides a valuable reference for leaf-heading research in other vegetables with leafy head, such as cabbage, mustard, lettuce, and endive, and the identification of the key transition leaves may be a valuable starting point to understand leaf head formation. However, the transition leaves may not be the exact leaves L7 identified by our study due to different species, different growth conditions, and even different heading types of the same species. In addition, our comprehensive and spatial transcriptome data cover almost all the aerial parts of Chinese cabbage at the heading stage. These data can be used to study leaf morphology and development, as well as to analyze the synthesis and accumulation of nutrient substances in leafy heads, such as glucosinolate, and explore the differentiation between homeologs after whole genome triplication.

## Materials and Methods

### Plant Materials and Sample Collection

Chinese cabbage (*B. rapa* cv Chiifu-401-42) was sown in potted trays in a greenhouse at August. After 3 weeks, seedlings were transplanted in the field with plastic sheds at the Chinese Academy of Agricultural Sciences. Eleven-week-old plants were used for sampling. Two normal growing Chinese cabbages were randomly selected. Since all head leaves arise on the axis of the enlarged but compressed stem, the spiral path of leaves may have five leaves in two whorls or eight leaves in three whorls (Zhang et al., 2021). The leaves in the same whorl showed similar size and similar morphology. Accordingly, all head leaves (leaf length > 2 cm) from the outer leaves to inner leaves were separated and collected in order, while the young and small inner leaves (leaf length < 2 cm) with the SAM were not separated and were collected as a mixed sample. A total of 30 leaves were obtained from each head. From the inside to the outside, every three leaves with similar morphology and similar size were taken as a whorl of leaves and a total of 10 whorls of leaves were obtained (Figure 1A). One leaf from every whorl was selected, named L1–L10, but only SAM, L1, L2, L3, L5, L7, and L9 were chosen for transcriptome analysis. L2 was dissected into two parts, leaf petiole (L2R2) and leaf blade (L2R1) for RNA-seq, while for L3, L5, L7, and L9, five regions, including the top (R1), outer margin (R2), and middle region (R3) of the blade and the top (R4) and middle (R5) regions of the petiole, were sampled. After sampling, a total of 48 samples from two biological replicates (two leafy heads) were ultimately used for transcriptome sequencing via the Illumina platform.

### RNA Isolation and Sequencing

Total RNA was extracted using the TRIzol reagent (Invitrogen, CA, United States). RNA integrity was confirmed using the 2100 Bioanalyzer. RNA-seq libraries were constructed according to the manufacturer’s protocol of the Vazyme mRNA-seq library preparation kit (Vazyme) and were sequenced to generate 150-nucleotide paired-end reads on a HiSeq platform (Illumina).

### Read Mapping and Differential Expression

The Chiifu reference genome (Brapa_v1.5; Cheng et al., 2016) was downloaded from http://brassicadb.org/brad/. After removing low-quality reads using the NGS QC Toolkit_v2.3.3 (Patel and Jain, 2012), clean reads were mapped to the Brapa_v1.5 reference genome using Hisat2-2.1.0 (Kim et al., 2015) with default settings for parameters. The bam files of uniquely mapped reads were used as inputs for the StringTie v1.3.4 software (Pertea et al., 2016), and TPM values were calculated to measure the expression levels of genes. To reduce transcription noise, only the genes with an average TPM ≥ 1 between two biological replicates were considered to be expressed and used for subsequent analysis.

Based on TPM values, DEGs were detected with a Fisher exact test (P-value cutoff < 0.05 and log2 fold change > 1 or <−1). By comparing the expression levels of genes at the corresponding positions of adjacent leaf blades (R1 vs R1, R2 vs R2, and R3 vs R3); that is, if a gene was upregulated or downregulated in multiple comparisons, it was considered a differential gene among leaf blades. SAM or HL1 were also used to compare adjacent blades. A similar method was also used to identify DEGs among petioles. The DEGs were identified between leaf blades and petioles by comparing the expression levels of genes in leaf blades and petioles from the same leaf; that is, if the expression level of a gene in any leaf blade sample was upregulated or downregulated relative to any petiole samples, it was considered to be a differential gene between leaf blades and petioles. Pearson correlation coefficient was calculated between biological replicates with the normalized expression levels of log2 (TPM value +1). Hierarchical clustering and PCA analysis were performed using the prcomp and hclust functions in R software (R Core Team, 2013) with default settings. The syntenic genes between *A. thaliana* and *B. rapa* were identified as previously described (Liang et al., 2016). An orthologous gene dataset was established for obtaining functional annotations of *B. rapa* genes based on *A. thaliana* genes.

### Co-expression Cluster Identification and GO Enrichment Analysis

Co-expression analysis was performed on all samples using MeV (V 4.9) with the k-means method (Gasch and Eisen, 2002). The normalized expression values of genes with Z-scores were used for k-Means clustering. Gene ontology enrichment analysis of each co-expression cluster was performed using agriGO with the singular enrichment analysis method (FDR < 0.05) (Tian et al., 2017).

### Content Assay of Soluble Sugars

The leaf blade or petiole samples from the same leaf layer were mixed together and ground in liquid nitrogen. Subsequently, 600 μl water and 800 μl internal standard (10 mg/ml arabinose water) were added to 200 mg ground powder, vortexed and mixed well. After sonication and of centrifugation for 10 min each, the mixture was filtered through a 0.22 μm filter membrane. Then, 895 μl of acetonitrile and 5 μl 20% ammonia water were added to 100 μl of filtrate, and the mixture was shaken to mix. The samples were maintained at in 4°C for 10 h and then filtered with a 0.22 μm organic membrane filter. The sugar content was then determined by UPLC-MS/MS (ACQUITY UPLC I-Class-Xevo TQ-S Micro, Waters).

### Co-expression Network Analysis

To identify modules of highly correlated genes, those genes with an average TPM of more than 1 were used to perform the co-expression network analysis by WGCNA. The one-step network construction and module detection were performed using the function blockwiseModules, with the following settings: the soft threshold power was set to 16, the maxBlockSize was set to 30,000, the minModuleSize was set to 30, and a dynamic tree cutoff was 0.25.

## Data Availability Statement

The datasets presented in this study can be found in online repositories. The names of the repository/repositories and accession number(s) can be found below: NCBI with the accession number PRJNA778186 (https://www.ncbi.nlm.nih.gov/sra?linkname=bioproject_sra_all&from_uid=778186).

## Author Contributions

XW, JW, and JL designed the study. JL and XG grew and collected the plant materials and performed the content assay of soluble sugars and morphological observation. XG and RL analyzed the RNA-seq data. XG, JL, and XW wrote the manuscript, with the help from JW and RL. All authors have discussed the results and approved the manuscript.

## Conflict of Interest

The authors declare that the research was conducted in the absence of any commercial or financial relationships that could be construed as a potential conflict of interest.

## Publisher’s Note

All claims expressed in this article are solely those of the authors and do not necessarily represent those of their affiliated organizations, or those of the publisher, the editors and the reviewers. Any product that may be evaluated in this article, or claim that may be made by its manufacturer, is not guaranteed or endorsed by the publisher.

## Supplementary Material

Supplementary Figure 1 Pearson correlation coefficients of transcriptome data and distribution of TPM normalized expression. **(A)** Pearson correlation coefficients of transcriptome data from biological replicates for 24 leaf tissues. **(B)** Distribution of TPM normalized expression among 24 leaf tissues.

Supplementary Figure 2 Distribution of expressed genes. **(A)** The numbers and proportion of expressed genes in 24 leaf tissues. **(B)** Distribution of expressed genes among different leaves.

Supplementary Figure 3 Distribution of DEGs among different leaves/tissue-types. **(A)** DEGs overlap for the blades of neighboring leaf. **(B)** DEGs overlap for the petioles of neighboring leaf. **(C)** Overlap of DEGs between leaf blades and petioles.

Supplementary Figure 4 K-means clustering grouped 9133 DEGs into 14 clusters. The X axis depicts 24 samples, and the Y axis represents the expression data per gene. For each gene, expression data were Z-score standardized.

Supplementary Figure 5 Cell proliferation and cell expansion genes constitute a regulatory network that controls leaf size.

Supplementary Figure 6 WGCNA of all expressed genes. **(A)** Hierarchical cluster tree showing co-expression modules identified by WGCNA. **(B)** Module–sample association. Each row corresponds to a module, labeled with a color as in panel **(A)**. Each column corresponds to a specific tissue. **(C)** Expression profiles of the 17 module eigengenes in all tissues. The X axis indicates the tissues; The Y axis indicates the value of the module eigengene.

Supplementary Figure 7 Expression pattern of some important genes identified in key transition leaves. **(A)** Expression profiles of the genes related to protein phosphorylation identified in KTLs. Vertical shade boxes indicate the position of key transition leaves. **(B)** Many auxin signaling genes were up-regulated in key transition leaves. Vertical shade boxes indicate the position of key transition leaves.

Supplementary Figure 8 Expression pattern of some important genes in transition leaves. **(A)** Many light-responsive genes were identified in key transition leaves. Vertical shade boxes indicate the position of key transition leaves. **(B)** Many genes encoding transcription factors were up-regulated in key transition leaves. Vertical shade boxes indicate the position of key transition leaves.

## References

[B1] Baba A. I. Andrási N. Valkai I. Gorcsa T. Koczka L. Darula Z. (2019). AtCRK5 protein kinase exhibits a regulatory role in hypocotyl hook development during skotomorphogenesis. *Int. J. Mol. Sci*. 20:3432. 10.3390/ijms20143432 PMC6678082 31336871

[B2] Baba A. I. Rigó G. Ayaydin F. Rehman A. U. Andrási N. Zsigmond L. (2018). Functional analysis of the *Arabidopsis thaliana* CDPK-related kinase family: At*CRK1* regulates responses to continuous light. *Int. J. Mol. Sci.* 19:1282. 10.3390/ijms19051282 29693594 PMC5983578

[B3] Cai X. Chang L. Zhang T. Chen H. Zhang L. Lin R. (2021). Impacts of allopolyploidization and structural variation on intraspecific diversification in *Brassica rapa*. *Genome Biol*. 22:166. 10.1186/s13059-021-02383-2 34059118 PMC8166115

[B4] Carles C. C. Fletcher J. C. (2003). Shoot apical meristem maintenance: the art of a dynamic balance. *Trends Plant Sci*. 8 394–401. 10.1016/S1360-1385(03)00164-X 12927973

[B5] Chen L. Q. Qu X. Q. Hou B. H. Sosso D. Osorio S. Fernie A. R. (2012). Sucrose efflux mediated by SWEET proteins as a key step for phloem transport. *Science* 335 207–211. 10.1126/science.1213351 22157085

[B6] Cheng F. Sun R. Hou X. Zheng H. Zhang F. Zhang Y. (2016). Subgenome parallel selection is associated with morphotype diversification and convergent crop domestication in *Brassica rapa* and *Brassica oleracea*. *Nat. Genet*. 48 1218–1224. 10.1038/ng.3634 27526322

[B7] Cutler S. R. Rodriguez P. L. Finkelstein R. R. Abrams S. R. (2010). Abscisic acid: emergence of a core signaling network. *Annu. Rev. Plant Biol*. 61 651–679. 10.1146/annurev-arplant-042809-112122 20192755

[B8] Ding G. Che P. Ilarslan H. Wurtele E. S. Nikolau B. J. (2012). Genetic dissection of methylcrotonyl CoA carboxylase indicates a complex role for mitochondrial leucine catabolism during seed development and germination. *Plant J*. 70 562–577. 10.1111/j.1365-313X.2011.04893.x 22211474

[B9] Ding Z. Galván-Ampudia C. S. Demarsy E. Łangowski Ł Kleine-Vehn J. Fan Y. (2011). Light-mediated polarization of the PIN3 auxin transporter for the phototropic response in *Arabidopsis*. *Nat. Cell Biol.* 13 447–452. 10.1038/ncb2208 21394084

[B10] Doering A. Lathe R. Persson S. (2012). An update on xylan synthesis. *Mol. Plant* 5 769–771. 10.1093/mp/sss049 22628543

[B11] Du F. Guan C. Jiao Y. (2018). Molecular mechanisms of leaf morphogenesis. *Mol. Plant* 11 1117–1134. 10.1016/j.molp.2018.06.006 29960106

[B12] Fu Q. Cheng L. Guo Y. Turgeon G. R. (2011). Phloem loading strategies in relation to water relations in trees and herbaceous plants. *Plant Physiol*. 157 1518–1527. 10.1104/pp.111.184820 21873572 PMC3252136

[B13] Gao L. Lyu S. W. Tang J. Zhou D. Bonnema G. Xiao D. (2017). Genome-wide analysis of auxin transport genes identifies the hormone responsive patterns associated with leafy head formation in Chinese cabbage. *Sci. Rep*. 7:42229. 10.1038/srep42229 28169368 PMC5294403

[B14] Gao Y. Huang S. Qu G. Fu W. Zhang M. Liu Z. (2020). The mutation of ent-kaurene synthase, a key enzyme involved in gibberellin biosynthesis, confers a non-heading phenotype to Chinese cabbage (*Brassica rapa* L. ssp. *pekinensis*). *Hortic. Res*. 7:178. 10.1038/s41438-020-00399-6 33328441 PMC7603516

[B15] Gasch A. P. Eisen M. B. (2002). Exploring the conditional coregulation of yeast gene expression through fuzzy k-means clustering. *Genome Biol*. 3:research0059. 10.1186/gb-2002-3-11-research0059 12429058 PMC133443

[B16] Gottwald J. R. Krysan P. J. Young J. C. Evert R. F. Sussman M. R. (2000). Genetic evidence for the in *planta* role of phloem-specific plasma membrane sucrose transporters. *Proc. Natl. Acad. Sci. U. S. A.* 97 13979–13984. 10.1073/pnas.250473797 11087840 PMC17686

[B17] Gu A. Meng C. Chen Y. Wei L. Dong H. Lu Y. (2017). Coupling Seq-BSA and RNA-Seq analyses reveal the molecular pathway and genes associated with heading type in Chinese cabbage. *Front. Genet*. 8:176. 10.3389/fgene.2017.00176 29312432 PMC5733010

[B18] Han C. Ren C. Zhi T. Zhou Z. Liu Y. Chen F. (2013). Disruption of fumarylacetoacetate hydrolase causes spontaneous cell death under short-day conditions in Arabidopsis. *Plant Physiol*. 162 1956–1964. 10.1104/pp.113.216804 23743712 PMC3729774

[B19] He Y. K. Xue W. X. Sun Y. D. Yu X. H. Liu P. L. (2000). Leafy head formation of the progenies of transgenic plants of Chinese cabbage with exogenous auxin genes. *Cell Res*. 10 151–160. 10.1038/sj.cr.7290044 10896176

[B20] Horiguchi G. Ferjani A. Fujikura U. Tsukaya H. (2006). Coordination of cell proliferation and cell expansion in the control of leaf size in *Arabidopsis thaliana*. *J. Plant Res*. 119 37–42. 10.1007/s10265-005-0232-4 16284709

[B21] Hou H. Erickson J. Meservy J. Schultz E. A. (2010). FORKED1 encodes a PH domain protein that is required for PIN1 localization in developing leaf veins. *Plant J*. 63 960–973. 10.1111/j.1365-313X.2010.04291.x 20626652

[B22] Huang H. Liu B. Liu L. Song S. (2017). Jasmonate action in plant growth and development. *J. Exp. Bot*. 68 1349–1359. 10.1093/jxb/erw495 28158849

[B23] Ito H. Kato T. (1957). Studies on the head formation of Chinese cabbage: histological and physiological studies of head formation. *J. Jpn. Soc. Hortic. Sci*. 26 154–162. 10.2503/jjshs.26.154

[B24] Jenness M. K. Tayengwa R. Murphy A. S. (2020). An ATP-binding cassette transporter, ABCB19, regulates leaf position and morphology during phototropin1-mediated blue light responses. *Plant Physiol*. 184 1601–1612. 10.1104/pp.20.00223 32855213 PMC7608178

[B25] Jun J. H. Ha C. M. Fletcher J. C. (2010). BLADE-ON-PETIOLE1 coordinates organ determinacy and axial polarity in *Arabidopsis* by directly activating ASYMMETRIC LEAVES2. *Plant Cell* 22 62–76. 10.1105/tpc.109.070763 20118228 PMC2828709

[B26] Kalve S. De Vos D. Beemster G. T. (2014). Leaf development: a cellular perspective. *Front. Plant Sci.* 5:362. 10.3389/fpls.2014.00362 25132838 PMC4116805

[B27] Kim D. Langmead B. Salzberg S. L. (2015). HISAT: a fast spliced aligner with low memory requirements. *Nat. Methods* 12 357–360. 10.1038/nmeth.3317 25751142 PMC4655817

[B28] Kim H. Park P. J. Hwang H. J. Lee S. Y. Oh M. H. Kim S. G. (2006). Brassinosteroid signals control expression of the *AXR3/IAA17* gene in the cross-talk point with auxin in root development. *Biosci. Biotechnol. Biochem*. 70 768–773. 10.1271/bbb.70.768 16636440

[B29] Kim T. W. Michniewicz M. Bergmann D. C. Wang Z. (2012). Brassinosteroid regulates stomatal development by GSK3-mediated inhibition of a MAPK pathway. *Nature* 482 419–422. 10.1038/nature10794 22307275 PMC3292258

[B30] Lee J. S. Wang S. Sritubtim S. Chen J. G. Ellis B. E. (2009). Arabidopsis mitogen-activated protein kinase MPK12 interacts with the MAPK phosphatase IBR5 and regulates auxin signaling. *Plant J*. 57 975–985. 10.1111/j.1365-313X.2008.03741.x 19000167

[B31] Li J. Zhang X. Lu Y. Feng D. Gu A. Wang S. (2019). Characterization of non-heading mutation in heading Chinese cabbage (*Brassica rapa* L. ssp. *pekinensis*). *Front. Plant Sci*. 10:112. 10.3389/fpls.2019.00112 30809236 PMC6379458

[B32] Li Y. Fan Y. Jiao Y. Wu J. Zhang Z. Yu X. (2019). Transcriptome profiling of yellow leafy head development during the heading stage in Chinese cabbage (*Brassica rapa* subsp. *pekinensis*). *Physiol. Plant* 165 800–813. 10.1111/ppl.12784 29900559

[B33] Li Y. Liu Z. Wang Y. Ning Y. Xin X. Yang S. (2012). Identification of quantitative trait loci for yellow inner leaves in Chinese cabbage (*Brassica rap*a L. ssp. *pekinensis*) based on SSR and SRAP markers. *Sci. Hortic*. 133 10–17. 10.1016/j.scienta.2011.10.023

[B34] Liang J. Liu B. Wu J. Cheng F. Wang X. (2016). Genetic variation and divergence of genes involved in leaf adaxial-abaxial polarity establishment in *Brassica rapa*. *Front. Plant Sci*. 7:94. 10.3389/fpls.2016.00094 26904064 PMC4746309

[B35] Ling J. J. Li J. Zhu D. Deng X. W. (2017). Noncanonical role of Arabidopsis COP1/SPA complex in repressing BIN2-mediated PIF3 phosphorylation and degradation in darkness. *Proc. Natl. Acad. Sci. U. S. A.* 114 3539–3544. 10.1073/pnas.1700850114 28292892 PMC5380025

[B36] Liu Q. Li J. Liu W. (2021). Sugar accumulation and characterization of metabolizing enzyme genes in leafy head of Chinese cabbage (*Brassica campestris* L. ssp. *pekinensis*). *Hortic. Environ. Biotechnol*. 62 17–29. 10.1007/s13580-020-00294-y

[B37] Mao Y. Wu F. Yu X. Bai J. Zhong W. He Y. (2014). MicroRNA319a-targeted *Brassica rapa* ssp. pekinensis TCP genes modulate head shape in chinese cabbage by differential cell division arrest in leaf regions. *Plant Physiol*. 164 710–720. 10.1104/pp.113.228007 24351684 PMC3912100

[B38] Marhava P. Bassukas A. E. L. Zourelidou M. Kolb M. Moret B. Fastner A. (2018). A molecular rheostat adjusts auxin flux to promote root protophloem differentiation. *Nature* 558 297–300. 10.1038/s41586-018-0186-z 29875411

[B39] Meyer S. Lauterbach C. Niedermeier M. Barth I. Sjolund D. R. Sauer N. (2004). Wounding enhances expression of AtSUC3, a sucrose transporter from Arabidopsis sieve elements and sink tissues. *Plant Physiol*. 134 684–693. 10.1104/pp.103.033399 14739351 PMC344544

[B40] Mouchel C. F. Osmont K. S. Hardtke C. S. (2006). BRX mediates feedback between brassinosteroid levels and auxin signalling in root growth. *Nature* 443 458–461. 10.1038/nature05130 17006513

[B41] Nakamura A. Nakajima N. Goda H. Shimada Y. Hayashi K. Nozaki H. (2006). Arabidopsis *Aux/IAA* genes are involved in brassinosteroid-mediated growth responses in a manner dependent on organ type. *Plant J*. 45 193–205. 10.1111/j.1365-313X.2005.02582.x 16367964

[B42] Nakata M. Okada K. (2013). The leaf adaxial-abaxial boundary and lamina growth. *Plants* 2 174–202. 10.3390/plants2020174 27137371 PMC4844365

[B43] Opena R. T. Kuo C. G. Voon J. Y. (1998). *Breeding and seed production of Chinese cabbage in the tropics and subtropics.* Taiwan: Asian Vegetable Research and Development Centre

[B44] Park J. Y. Canam T. Kang K. Y. Ellis D. D. Mansfield S. D. (2008). Over-expression of an arabidopsis family A sucrose phosphate synthase (SPS) gene alters plant growth and fibre development. *Transgenic Res.* 17 181–192. 10.1007/s11248-007-9090-2 17415671

[B45] Patel R. K. Jain M. (2012). NGS QC Toolkit: a toolkit for quality control of next generation sequencing data. *PLoS One* 7:e30619. 10.1371/journal.pone.0030619 22312429 PMC3270013

[B46] Pattanayak G. K. Venkataramani S. Hortensteiner S. Kunz L. Christ B. Moulin M. (2012). Accelerated cell death 2 suppresses mitochondrial oxidative bursts and modulates cell death in *Arabidopsis*. *Plant J.* 69 589–600. 10.1111/j.1365-313X.2011.04814.x 21988537 PMC3274588

[B47] Pérez-Pérez J. M. Candela H. Robles P. López-Torrejón G. del Pozo J. C. Micol J. L. (2010). A role for AUXIN RESISTANT3 in the coordination of leaf growth. *Plant Cell Physiol*. 51 1661–1673. 10.1093/pcp/pcq123 20739302

[B48] Pertea M. Kim D. Pertea G. M. Leek J. T. Salzberg S. L. (2016). Transcript-level expression analysis of RNA-seq experiments with HISAT, StringTie and Ballgown. *Nat. Protoc*. 11 1650–1667. 10.1038/nprot.2016.095 27560171 PMC5032908

[B49] Powell A. Lenhard M. (2012). Control of organ size in plants. *Curr. Biol*. 22 360–367. 10.1016/j.cub.2012.02.010 22575478

[B50] R Core Team (2013). *R: A Language and Environment for Statistical Computing.* Vienna: R Foundation for Statistical Computing. Available online at: http://www.R-project.org

[B51] Ren W. Wu F. Bai J. Li X. Yang X. Xue W. (2020). BcpLH organizes a specific subset of microRNAs to form a leafy head in Chinese cabbage (*Brassica rapa* ssp. *pekinensis*). *Hortic. Res*. 7:1. 10.1038/s41438-019-0222-7 31908804 PMC6938484

[B52] Rodrigues C. M. Müdsam C. Keller I. Zierer W. Czarnecki O. Corral J. M. (2020). Vernalization alters sink and source identities and reverses phloem translocation from taproots to shoots in sugar beet. *Plant Cell* 32 3206–3223. 10.1105/tpc.20.00072 32769131 PMC7534467

[B53] Ruan Y. (2014). Sucrose metabolism: gateway to diverse carbon use and sugar signaling. *Annu. Rev. Plant Biol*. 65 33–67. 10.1146/annurev-arplant-050213-040251 24579990

[B54] Scacchi E. Osmont K. S. Beuchat J. Salinas P. Navarrete-Gómez M. Trigueros M. (2009). Dynamic, auxin-responsive plasma membrane-to-nucleus movement of *Arabidopsis* BRX. *Development* 136 2059–2067. 10.1242/dev.035444 19465596

[B55] Schneider S. Hulpke S. Schulz A. Yaron I. Höll J. Imlau A. (2012). Vacuoles release sucrose via tonoplast-localised SUC4-type transporters. *Plant Biol*. 14 325–336. 10.1111/j.1438-8677.2011.00506.x 21972845

[B56] Schulz A. Beyhl D. Marten I. Wormit A. Neuhaus E. Poschet G. (2011). Proton-driven sucrose symport and antiport are provided by the vacuolar transporters SUC4 and TMT1/2. *Plant J*. 68 129–136. 10.1111/j.1365-313X.2011.04672.x 21668536

[B57] Shi S. Li S. Asim M. Mao J. Xu D. Ullah Z. (2018). The Arabidopsis calcium-dependent protein kinases (CDPKs) and their roles in plant growth regulation and abiotic stress responses. *Int. J. Mol. Sci*. 19:1900. 10.3390/ijms19071900 29958430 PMC6073581

[B58] Shin A. Y. Han Y. J. Baek A. Ahn T. Kim S. Y. Nguyen T. S. (2016). Evidence that phytochrome functions as a protein kinase in plant light signalling. *Nat. Commun*. 7:11545. 10.1038/ncomms11545 27173885 PMC4869175

[B59] Sun X. Basnet R. K. Yan Z. Bucher J. Cai C. Zhao J. (2019). Genome-wide transcriptome analysis reveals molecular pathways involved in leafy head formation of Chinese cabbage (*Brassica rapa*). *Hortic. Res*. 6:130. 10.1038/s41438-019-0212-9 31814983 PMC6885048

[B60] Tian T. Liu Y. Yan H. You Q. Yi X. Du Z. (2017). agriGO v2.0: a GO analysis toolkit for the agricultural community, 2017 update. *Nucleic Acids Res.* 45 W122–W129. 10.1093/nar/gkx382 28472432 PMC5793732

[B61] Tsukaya H. (2003). Organ shape and size: a lesson from studies of leaf morphogenesis. *Curr. Opin. Plant Biol*. 6 57–62. 10.1016/s1369526602000055 12495752

[B62] Vernoux T. Kronenberger J. Grandjean O. Laufs P. Traas J. (2000). PIN-FORMED 1 regulates cell fate at the periphery of the shoot apical meristem. *Development* 127 5157–5165. 11060241 10.1242/dev.127.23.5157

[B63] Wang F. Li L. Li H. Liu L. Zhang Y. Gao J. (2012). Transcriptome analysis of rosette and folding leaves in Chinese cabbage using high-throughput RNA sequencing. *Genomics* 99 299–307. 10.1016/j.ygeno.2012.02.005 22387604

[B64] Wang X. Yu R. Wang J. Lin Z. Han X. Deng Z. (2020). The Asymmetric Expression of SAUR genes mediated by ARF7/19 promotes the gravitropism and phototropism of plant hypocotyls. *Cell Rep*. 31:107529. 10.1016/j.celrep.2020.107529 32320660

[B65] Wang Y. Wu F. Bai J. He Y. (2014). *BrpSPL9* (*Brassica rapa* ssp. *pekinensis SPL9*) controls the earliness of heading time in Chinese cabbage. *Plant Biotechnol. J*. 12 312–321. 10.1111/pbi.12138 24237584

[B66] Wasternack C. Song S. (2017). Jasmonates: biosynthesis, metabolism, and signaling by proteins activating and repressing transcription. *J. Exp. Bot*. 68 1303–1321. 10.1093/jxb/erw443 27940470

[B67] Xu J. Zhang S. (2015). Mitogen-activated protein kinase cascades in signaling plant growth and development. *Trends Plant Sci*. 20 56–64. 10.1016/j.tplants.2014.10.001 25457109

[B68] Yan Y. Li C. Dong X. Li H. Zhang D. Zhou Y. (2020). MYB30 is a key negative regulator of Arabidopsis photomorphogenic development that promotes PIF4 and PIF5 protein accumulation in the light. *Plant Cell* 32 2196–2215. 10.1105/tpc.19.00645 32371543 PMC7346557

[B69] Yang C. Zhang C. Lu Y. Jin J. Wang X. (2011). The mechanisms of brassinosteroids’ action: from signal transduction to plant development. *Mol. Plant* 4 588–600. 10.1093/mp/ssr020 21471332

[B70] Yu C. Yan C. Liu Y. Liu Y. Jia Y. Lavelle D. (2020). Upregulation of a *KN1* homolog by transposon insertion promotes leafy head development in lettuce. *Proc. Natl. Acad. Sci. U. S. A.* 117 33668–33678. 10.1073/pnas.2019698117 33288708 PMC7776633

[B71] Yu X. Peng J. Feng X. Yang S. Zheng Z. Tang X. (2000). Cloning and structural and expressional characterization of *BcpLH* gene preferentially expressed in folding leaf of Chinese cabbage. *Sci. China C Life Sci.* 43 321–329. 10.1007/BF02879292 18726388

[B72] Yu X. Wang H. Zhong W. Bai J. Liu P. He Y. (2013). QTL mapping of leafy heads by genome resequencing in the RIL population of *Brassica rapa*. *PLoS One* 8:e76059. 10.1371/journal.pone.0076059 24204591 PMC3810141

[B73] Zhang K. Wang X. Cheng F. (2019). Plant polyploidy: origin, evolution, and its influence on crop domestication. *Hortic. Plant J*. 5 231–239. 10.1016/j.hpj.2019.11.003

[B74] Zhang Y. Liang J. Cai X. Chen H. Wu J. Lin R. (2021). Divergence of three *BRX* homoeologs in *Brassica rapa* and its effect on leaf morphology. *Hortic. Res*. 8:68. 10.1038/s41438-021-00504-3 PMC8012600 33790228

