# Supplementary figures and images for: Series-Spatial Transcriptome Profiling of Leafy Head Reveals the Key Transition Leaves for Head Formation in Chinese Cabbage
Source: Front Plant Sci. 2022 Jan 6;12:787826. doi: 10.3389/fpls.2021.787826 (PMC8770947; doi:10.3389/fpls.2021.787826)

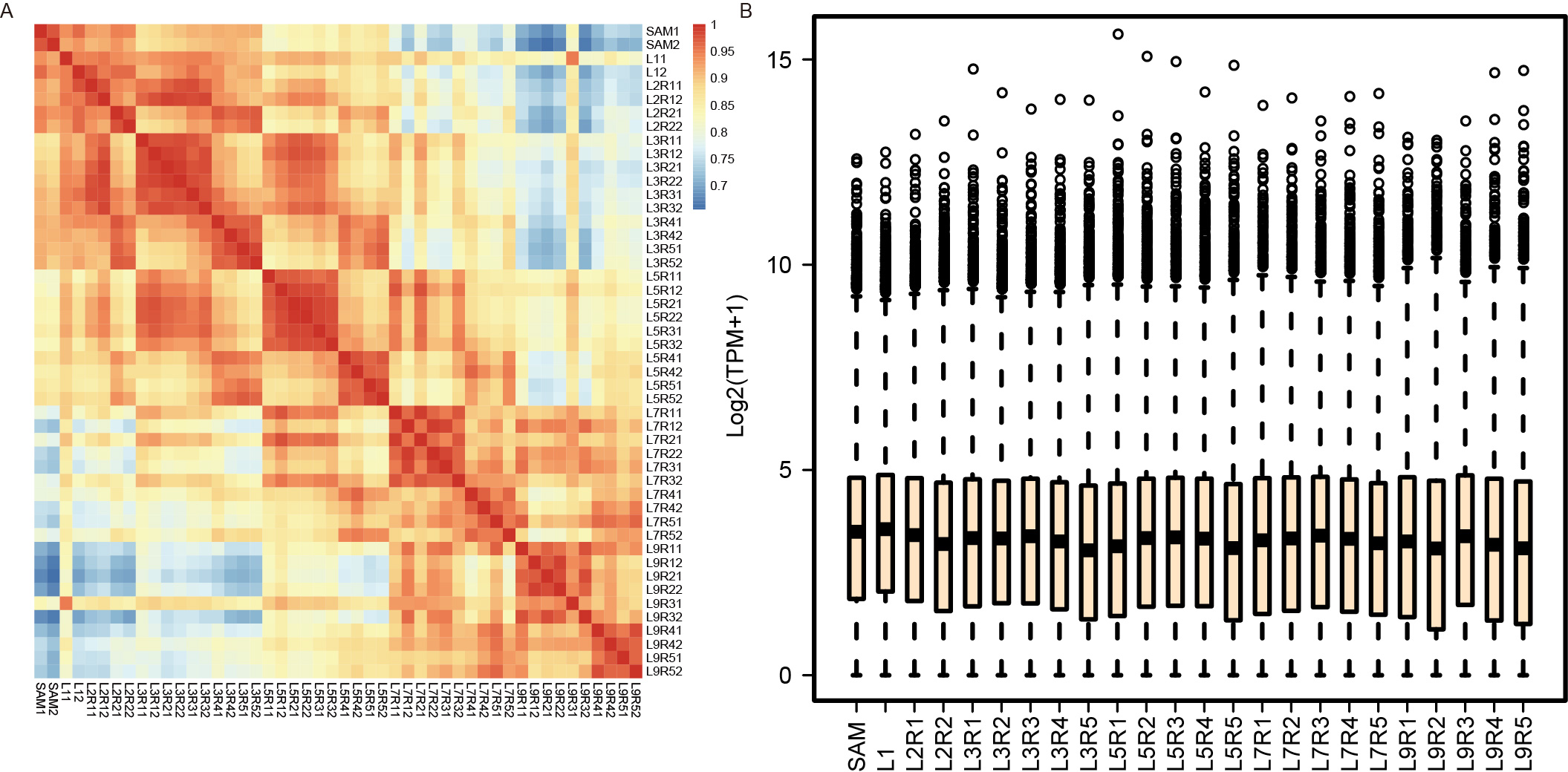

Supplement: Supplementary Figure 1 — Pearson correlation coefficients of transcriptome data and distribution of TPM normalized expression. (A) Pearson correlation coefficients of transcriptome data from biological replicates for 24 leaf tissues. (B) Distribution of TPM normalized expression among 24 leaf tissues. [file Image_1.JPEG]

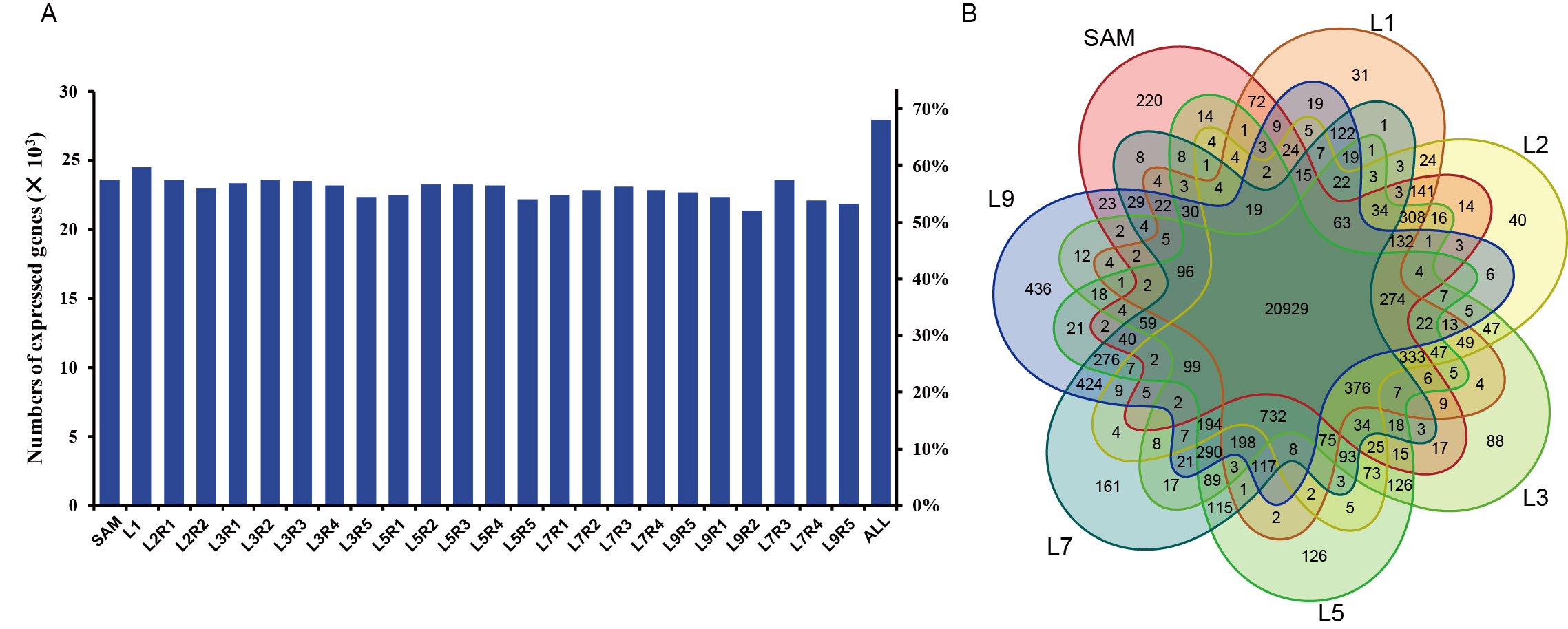

Supplement: Supplementary Figure 2 — Distribution of expressed genes. (A) The numbers and proportion of expressed genes in 24 leaf tissues. (B) Distribution of expressed genes among different leaves. [file Image_2.JPEG]

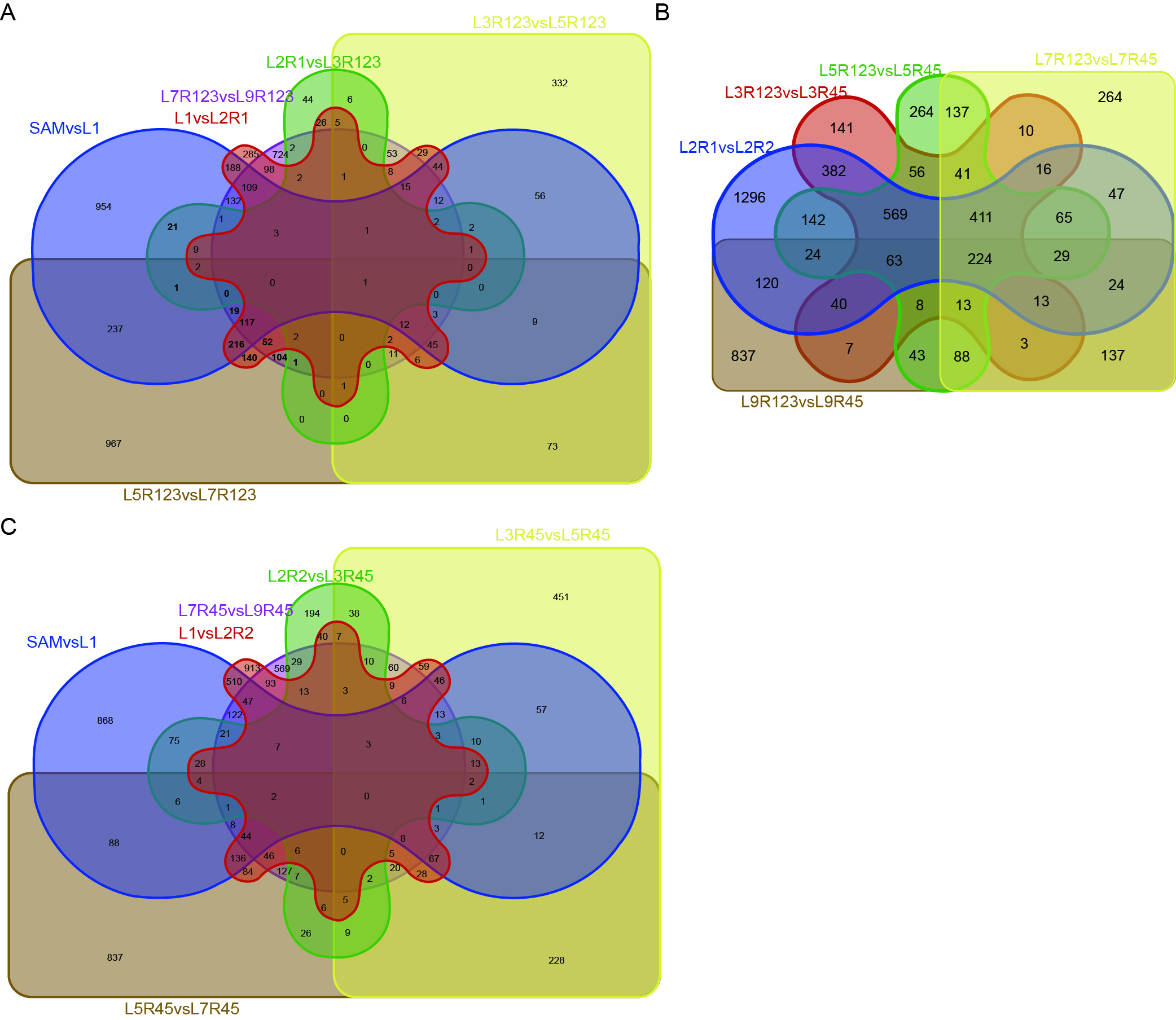

Supplement: Supplementary Figure 3 — Distribution of DEGs among different leaves/tissue-types. (A) DEGs overlap for the blades of neighboring leaf. (B) DEGs overlap for the petioles of neighboring leaf. (C) Overlap of DEGs between leaf blades and petioles. [file Image_3.JPEG]

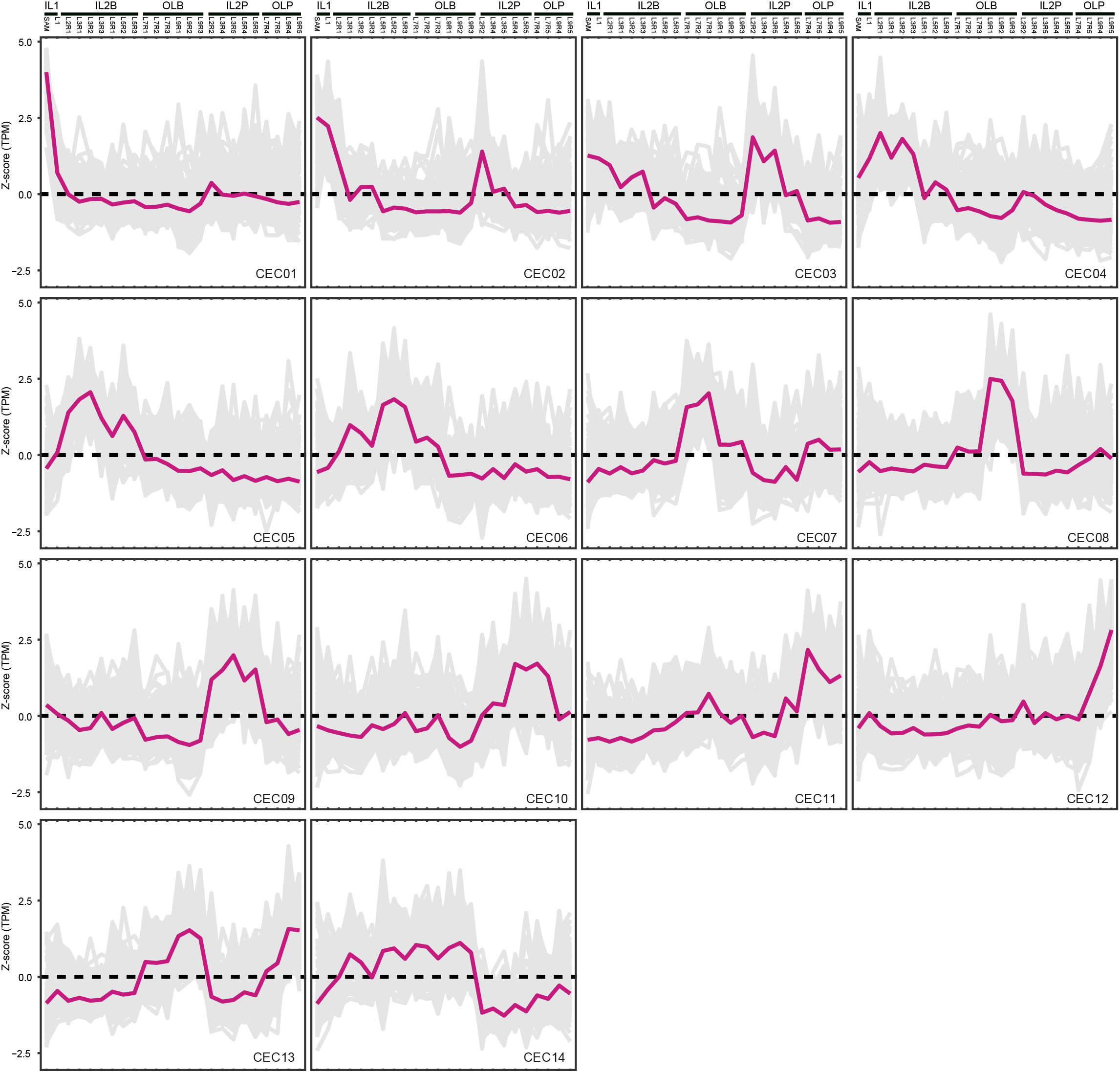

Supplement: Supplementary Figure 4 — K-means clustering grouped 9133 DEGs into 14 clusters. The X axis depicts 24 samples, and the Y axis represents the expression data per gene. For each gene, expression data were Z-score standardized. [file Image_4.JPEG]

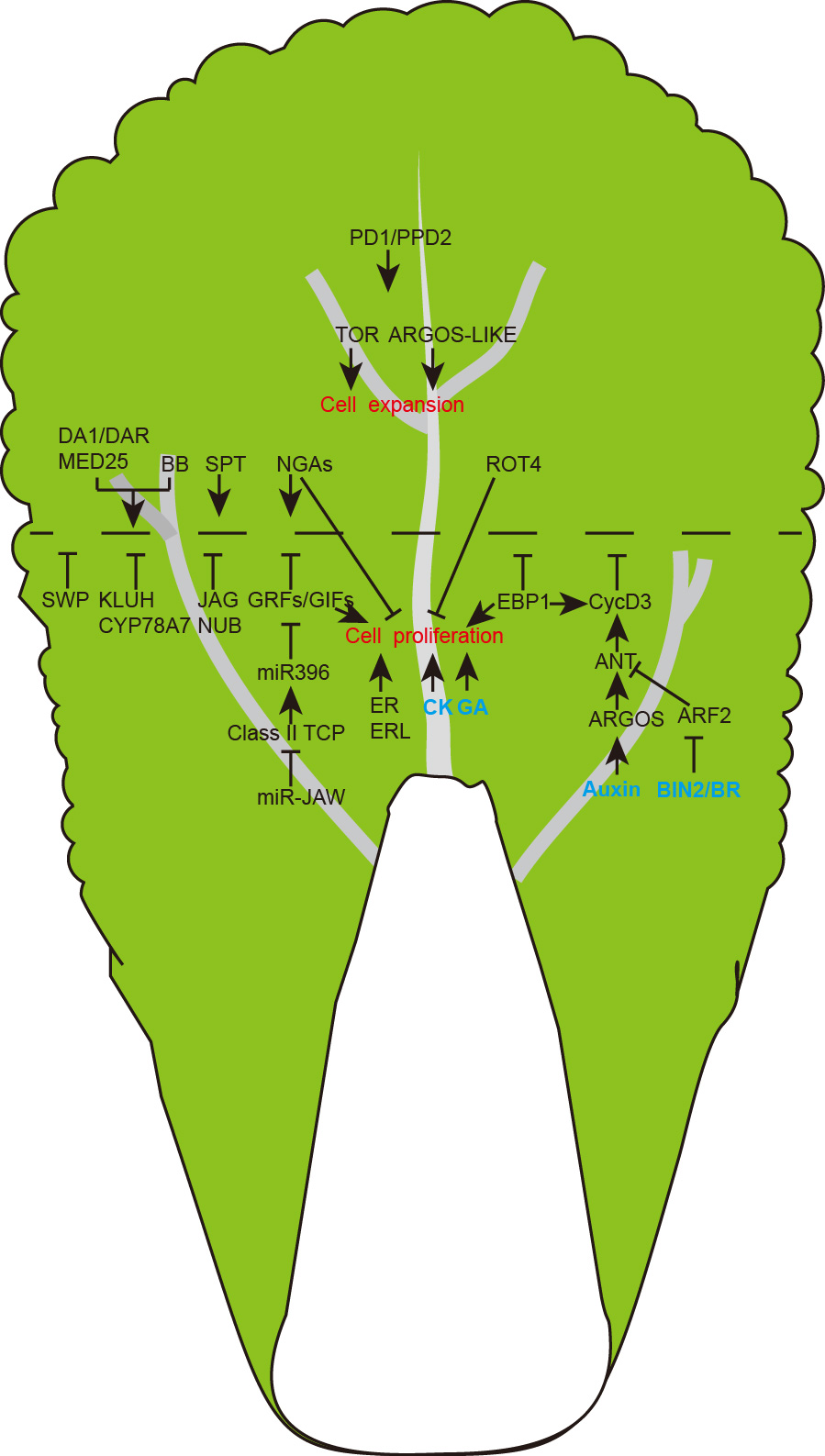

Supplement: Supplementary Figure 5 — Cell proliferation and cell expansion genes constitute a regulatory network that controls leaf size. [file Image_5.JPEG]

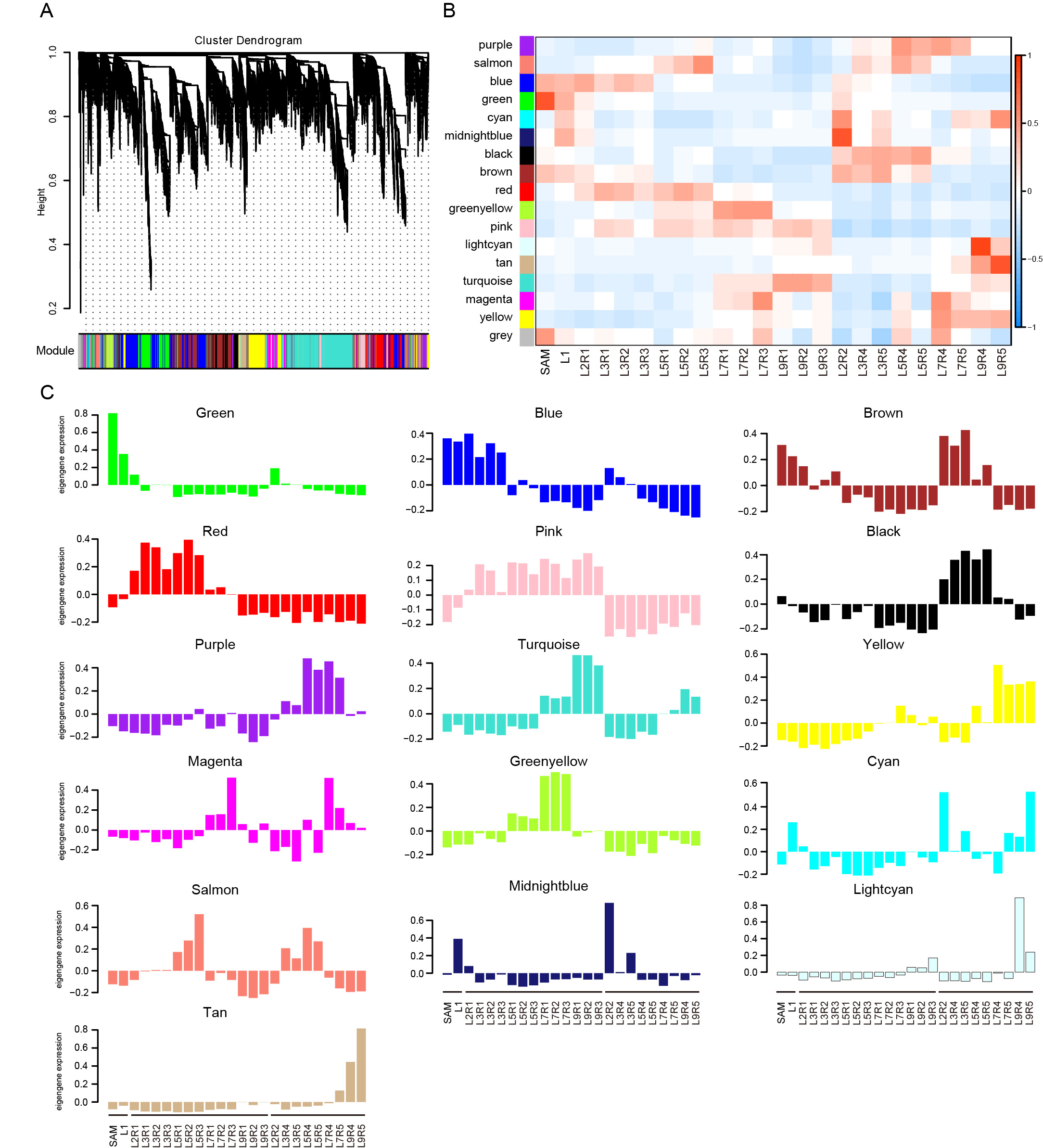

Supplement: Supplementary Figure 6 — WGCNA of all expressed genes. (A) Hierarchical cluster tree showing co-expression modules identified by WGCNA. (B) Module–sample association. Each row corresponds to a module, labeled with a color as in panel (A). Each column corresponds to a specific tissue. (C) Expression profiles of the 17 module eigengenes in all tissues. The X axis indicates the tissues; The Y axis indicates the value of the module eigengene. [file Image_6.JPEG]

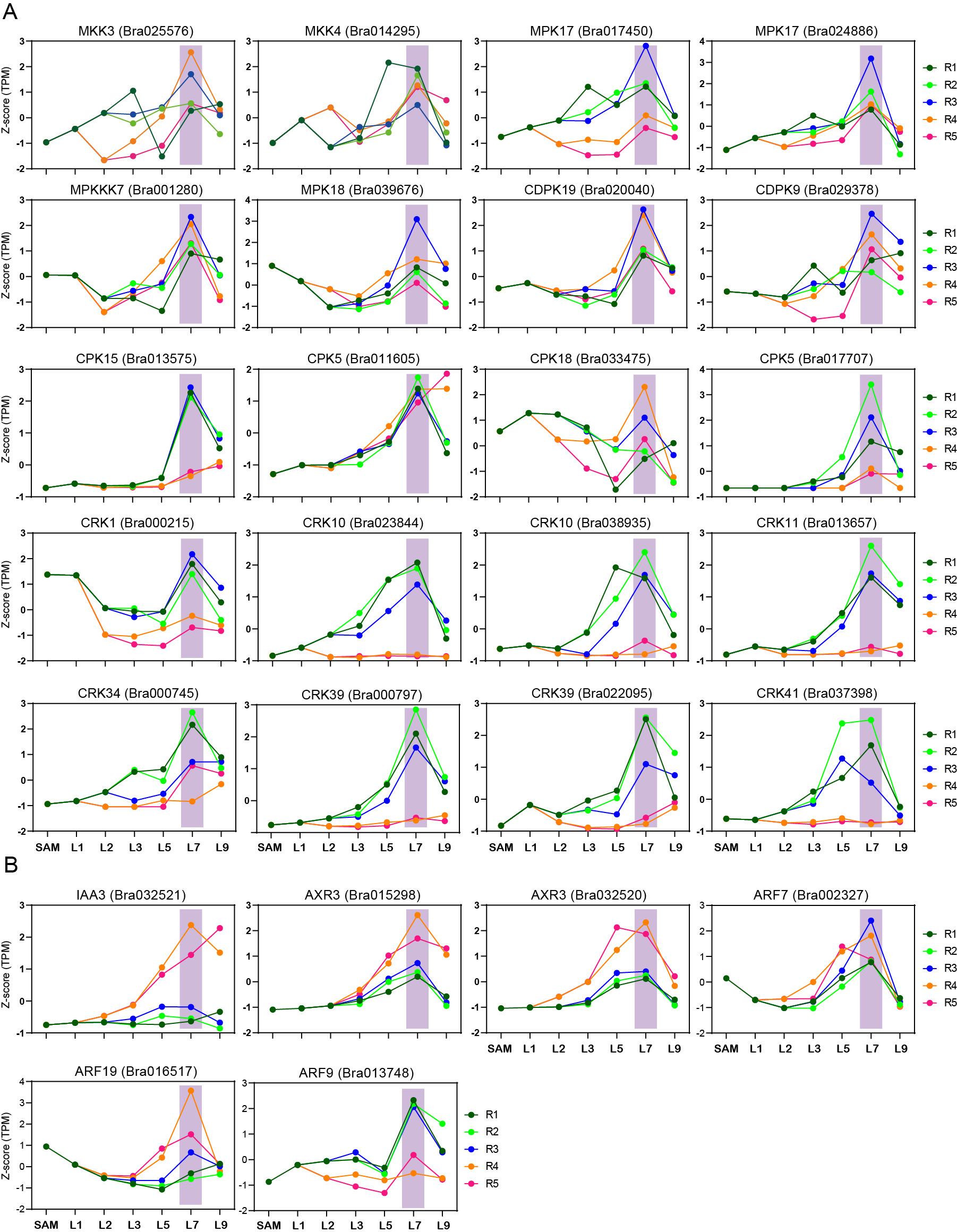

Supplement: Supplementary Figure 7 — Expression pattern of some important genes identified in key transition leaves. (A) Expression profiles of the genes related to protein phosphorylation identified in KTLs. Vertical shade boxes indicate the position of key transition leaves. (B) Many auxin signaling genes were up-regulated in key transition leaves. Vertical shade boxes indicate the position of key transition leaves. [file Image_7.JPEG]

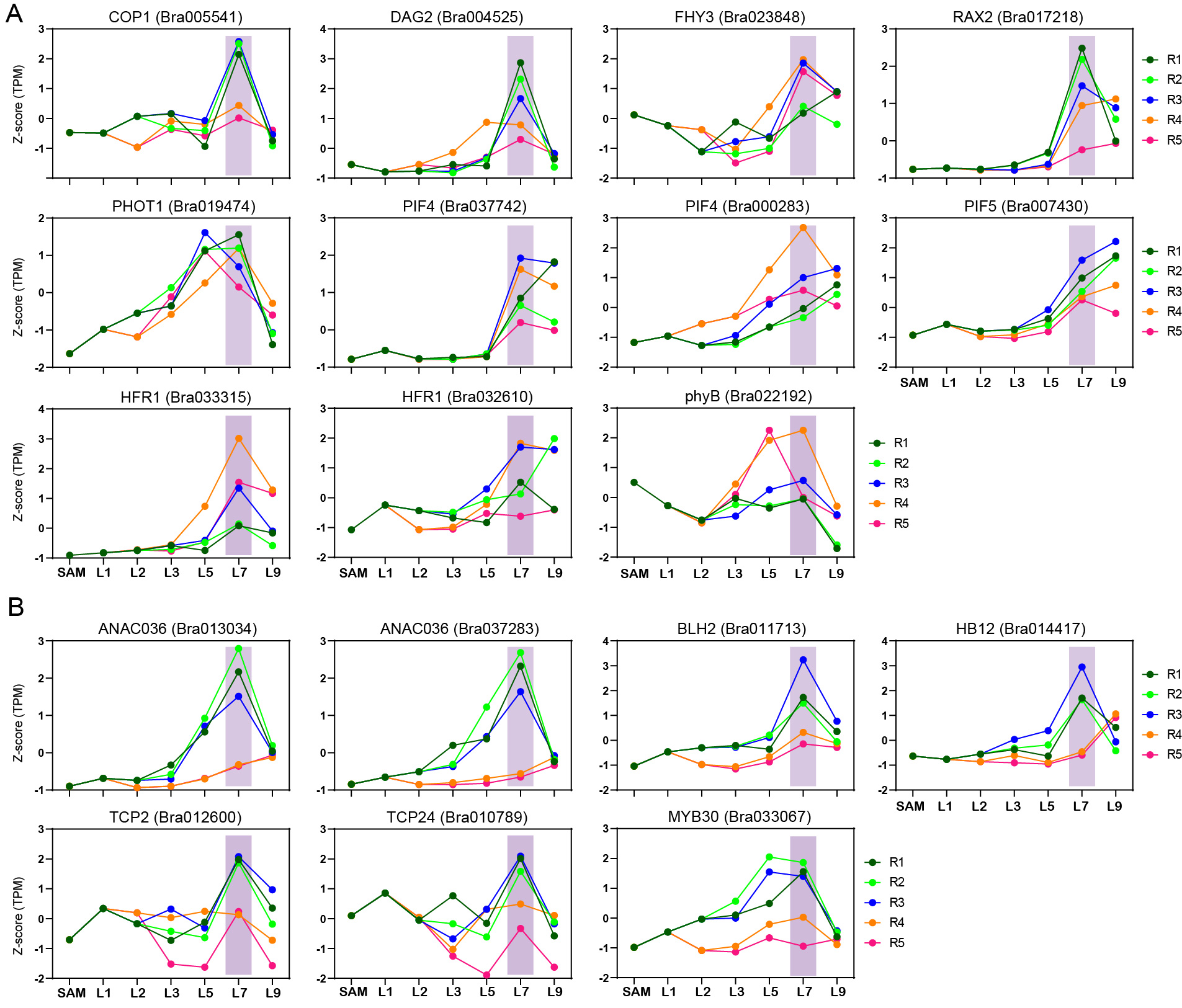

Supplement: Supplementary Figure 8 — Expression pattern of some important genes in transition leaves. (A) Many light-responsive genes were identified in key transition leaves. Vertical shade boxes indicate the position of key transition leaves. (B) Many genes encoding transcription factors were up-regulated in key transition leaves. Vertical shade boxes indicate the position of key transition leaves. [file Image_8.JPEG]
